# Downregulation of ERG and FLI1 expression in endothelial cells triggers endothelial-to-mesenchymal transition

**DOI:** 10.1371/journal.pgen.1007826

**Published:** 2018-11-30

**Authors:** Nao Nagai, Hiroto Ohguchi, Ryo Nakaki, Yoshihiro Matsumura, Yasuharu Kanki, Juro Sakai, Hiroyuki Aburatani, Takashi Minami

**Affiliations:** 1 Division of Molecular and Vascular Biology, IRDA, Kumamoto University, Kumamoto, Japan; 2 Graduate School of Pharmaceutical Sciences, The University of Tokyo, Tokyo, Japan; 3 Division of Disease Epigenetics, IRDA, Kumamoto University, Kumamoto, Japan; 4 Division of Genome Sciences, RCAST, The University of Tokyo, Tokyo, Japan; 5 Division of Metabolic Medicine, RCAST, The University of Tokyo, Tokyo, Japan; 6 Isotope Science Center, The University of Tokyo, Tokyo, Japan; Zeisberg lab, GERMANY

## Abstract

Endothelial cell (EC) plasticity in pathological settings has recently been recognized as a driver of disease progression. Endothelial-to-mesenchymal transition (EndMT), in which ECs acquire mesenchymal properties, has been described for a wide range of pathologies, including cancer. However, the mechanism regulating EndMT in the tumor microenvironment and the contribution of EndMT in tumor progression are not fully understood. Here, we found that combined knockdown of two ETS family transcription factors, *ERG* and *FLI1*, induces EndMT coupled with dynamic epigenetic changes in ECs. Genome-wide analyses revealed that ERG and FLI1 are critical transcriptional activators for EC-specific genes, among which microRNA-126 partially contributes to blocking the induction of EndMT. Moreover, we demonstrated that *ERG* and *FLI1* expression is downregulated in ECs within tumors by soluble factors enriched in the tumor microenvironment. These data provide new insight into the mechanism of EndMT, functions of ERG and FLI1 in ECs, and EC behavior in pathological conditions.

## Author summary

Differentiated cells possess unique characteristics to maintain vital activities. However, cells occasionally show abnormal behavior in pathological settings due to dysregulated gene expression. Endothelial-to-mesenchymal transition (EndMT) is a phenomenon in which endothelial cells lose their characteristics and acquire mesenchymal-like properties. Although EndMT is observed in various diseases including cancer, and augments fibrosis and vascular defects, the mechanism of EndMT induction is not fully understood. Here, we show that EndMT is triggered via reduced expression of *ERG* and *FLI1*, which have recently been recognized as pivotal transcription factors in endothelial cells (ECs). Mechanistically, ERG and FLI1 activate EC-specific genes and repress mesenchymal-like genes via epigenetic regulation to prevent EndMT. Furthermore, we demonstrate that microRNA-126, which is specifically expressed in ECs, is the key downstream target of ERG/FLI1 for regulating EndMT. Finally, we show that ERG and FLI1 expression is decreased in ECs within tumors, suggesting that EndMT is induced in the tumor microenvironment. Collectively, these findings indicate that loss of *ERG* and *FLI1* leads to the aberrant behavior of ECs in pathological conditions.

## Introduction

Reciprocal interactions between tumor cells and stromal cells have a profound impact on tumor progression. Among tumor stromal cells, endothelial cells (ECs) and blood vessels are important components, as they supply nutrients and oxygen, and act as an entrance into systemic circulation, leading to metastatic organs. However, the behavior of ECs within tumors is not fully understood. Endothelial-to-mesenchymal transition (EndMT) is a phenotypic conversion process in which ECs lose their specific characteristics and obtain mesenchymal properties. Whereas EndMT is physiologically induced during embryonic heart development [1, 2], it is also pathologically induced in a wide range of diseases associated with fibrosis and vasculopathy [3, 4]. In cancer pathology, a landmark study by Zeisberg *et al*. showed that EndMT is a potential source of cancer-associated fibroblasts (CAFs), which are well-known tumor stromal cells typically recognized to have a pro-tumor role [5, 6]. However, the mechanism of EndMT induction in the tumor microenvironment and the impact of EndMT on tumor progression remain unclear.

Our group previously reported that knockdown of GATA2, a transcription factor (TF) essential for EC differentiation and function, induces EndMT-like conversion in ECs, suggesting that dysregulation of EC-related TFs can trigger EndMT [7]. Interestingly, ECs have no single master regulator, and this role appears to be shared by a variety of EC-related TFs, including the ETS, GATA, SOX, and FOX families [8]. A strong candidate for a pioneer TF in ECs, which appears at the earliest stage of cell fate determination and binds closed chromatin to form lineage-specific epigenetic conditions, is ETV2, a member of the ETS family. However, *ETV2* is expressed transiently during EC differentiation and is not detected in mature ECs [9, 10]. Thus, ETS factors specifically expressed in mature ECs, ERG and FLI1, may be especially important among EC-related TFs. This is supported by recent data showing that constitutive expression of *ERG* and *FLI1* with transient expression of *ETV2* directly reprograms amniotic cells to mature ECs [11].

The ETS family is a member of TFs with a well-conserved DNA binding domain named the ETS domain, which typically recognizes the consensus sequence 5′-GGAA-3′ [12]. Among them, ETS-related gene (ERG) and Friend leukemia integration 1 (FLI1) are specifically and highly expressed in ECs [13]. Notably, *ERG* and *FLI1* show ~70% overall amino acid sequence similarity and only 2 mismatches within ~80 amino-acid stretch in the ETS domain. *ERG*- and *FLI1*-knockout mice commonly show embryonic lethality at E10.5–11.5 and severe hemorrhage due to defective angiogenesis [14–17]. In addition, double knockdown of *erg* and *fli1* in zebrafish showed more severe vascular defects compared to individual knockdown, indicating that these TFs have synergistic roles [18]. In support of this, several groups demonstrated that ERG promotes the expression of some EC-specific genes such as *CDH5*, *HDAC6*, *CLDN5*, *ENG*, *VWF*, and *RHOJ* to maintain EC function, while *ENG* is also under the control of FLI1 [19–25]. Additionally, a recent paper reported that ERG controls the TGFβ/SMAD signaling pathway to protect ECs from EndMT [26]. Although the emerging roles of ERG and FLI1 have been recognized in ECs, the functions of these TFs have not been thoroughly analyzed using genome-wide approaches.

In this study, we conducted a comprehensive microarray and chromatin immunoprecipitation-sequencing (ChIP-seq) analysis to characterize the ERG- and FLI1-mediated transcriptional regulation in ECs. Our results indicate that combined downregulation of *ERG* and *FLI1* expression leads to EndMT associated with dynamic changes in transcriptome and epigenome. We identified microRNA-126, which is specifically expressed in ECs, as the key downstream target of ERG and FLI1 to regulate EndMT. Furthermore, we also show that *ERG* and *FLI1* expression is downregulated in tumor tissues by soluble factors. These findings might provide new insight into EC phenotypic changes mediated by the loss of ERG/FLI1 in the pathological environment.

## Results

### Combined knockdown of *ERG* and *FLI1* induces EndMT

**Fig 1:**
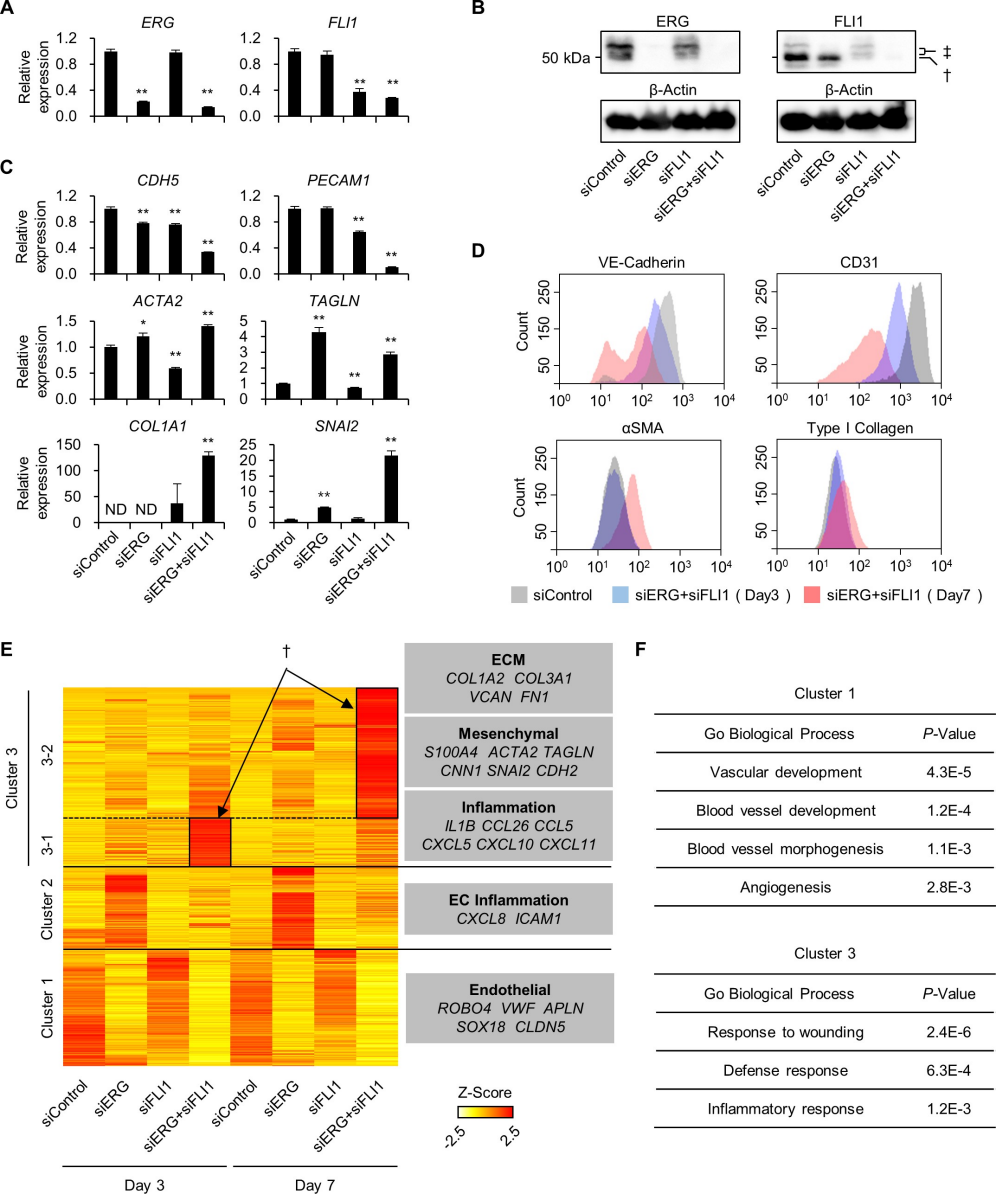
Combined knockdown of *ERG* and *FLI1* induces EndMT. (A) Relative expression of *ERG* and *FLI1* quantified by qPCR in HUVECs treated with siERG, siFLI1, or both for 3 days. Data are represented as mean ± SEM (n = 3–4). *P < 0.05; **P < 0.01 by Student’s *t*-test. (B) Immunoblot analysis of ERG and FLI1 in HUVECs treated with siERG, siFLI1, or both for 3 days. †, FLI1; ‡, ERG detected because of anti-FLI1 antibody cross-reactivity. See also S4A Fig. (C) Relative expression of endothelial/mesenchymal markers quantified by qPCR in HUVECs treated with siERG, siFLI1, or both for 3 days. Data are represented as mean ± SEM (n = 4). *P < 0.05; **P < 0.01 by Student’s *t*-test. ND, not detected. (D) Flow cytometry analysis of endothelial/mesenchymal marker expression in HUVECs treated with siERG+siFLI1 for 3 or 7 days. (E) Heatmap of 1,190 differentially expressed genes determined by microarray in HUVECs treated with siERG, siFLI1, or both for 3 or 7 days. Representative genes in each cluster are listed on the right. Genes synergistically repressed by ERG and FLI1 are marked with a dagger (†). (F) Gene ontology terms enriched in clusters 1 and 3 are listed. See also S1–S3 Figs.

Considering previous findings that constitutive *ERG* and *FLI1* expression with transient *ETV2* expression directly reprograms somatic cells into ECs, we assessed how the ablation of *ERG* and/or *FLI1* affects mature EC phenotype [11]. We knocked down these TFs either alone or in combination using two independent sets of siRNAs in primary cultured human umbilical vein ECs (HUVECs) (Fig 1A and 1B and S1A Fig). Consistent with the previous report that depletion of ERG expression leads to EndMT [26], knockdown of ERG alone upregulated a mesenchymal marker (*TAGLN*) and an epithelial-to-mesenchymal transition (EMT)/EndMT driver gene (*SNAI2*). In contrast, knockdown of FLI1 alone did not upregulate these EndMT marker expression. Interestingly, combined knockdown of *ERG* and *FLI1* using 2 sets of siRNAs effectively led to decreased expression of endothelial markers (*CDH5*, *PECAM1*) and increased expression of EndMT markers (*ACTA2*, *TAGLN*, *COL1A1*, *and SNAI2*) consistently (Fig 1C and S1B Fig). EndMT-like conversion was also observed at the protein level, with an indication that the conversion process requires 7 days of culture to be completed (Fig 1D). Along with marker expression changes, HUVECs morphologically changed from an EC-specific cobblestone-like shape into a mesenchymal-like spindle shape (S1C Fig). Additionally, combined knockdown of *ERG* and *FLI1* led to a defect in EC function as indicated by the loss of tube formation ability (S1D Fig). Given that reduced expression of *ERG* triggers apoptosis [20], we evaluated the apoptosis level by cleaved caspase-3 immuno-detection after siRNA treatment targeting *ERG* and *FLI1* individually and together. Combined treatment of siERG and siFLI1 as well as siERG treatment alone significantly induced caspase-3 cleavage (S1E Fig). However, cleaved caspase-3 comprised a minor fraction, indicating that apoptotic cell death does not mainly affect the cell phenotype as a whole in our experiments. To confirm the induction of EndMT in a genome-wide manner, transcriptomic changes were analyzed in HUVECs treated with siERG, siFLI1, or both, for 3 or 7 days using a gene expression microarray (Fig 1E). We identified 1,190 differentially expressed genes, which were classified into 3 clusters: cluster 1, genes driven by ERG and/or FLI1; cluster 2, genes repressed by ERG alone; and cluster 3, genes synergistically repressed by ERG and FLI1. Cluster 3 was further classified into two sub-clusters: cluster 3–1, genes upregulated 3 days after siRNA treatment; and cluster 3–2, genes upregulated 7 days after siRNA treatment.

Cluster 1 includes EC specific genes coding for *ROBO4*, *vWF*, *Apelin*, *SOX18*, and *Claudin-5* (Fig 1E). Gene ontology (GO) analysis confirmed that this cluster is highly related to vascular functions including ‘vascular development’ and ‘angiogenesis’ (Fig 1F). Importantly, cluster 1 genes were reduced further by siERG than by siFLI1, but significantly further reduced by siERG and siFLI1 in combination compared to siERG alone (S2A Fig). Cluster 2 includes genes encoding *IL-8* and *ICAM1* (Fig 1E), consistent with the previous reports showing that these genes are upregulated by *ERG* knockdown [27, 28]. GO analysis showed an enrichment of genes related to mitosis and cell cycle in cluster 2 (S2C Fig). In contrast, cluster 3 is comprised of mesenchymal-related genes encoding mesenchymal markers (S100A4/FSP-1, αSMA, SM-22α, and N-Cadherin), extracellular matrix (ECM) (collagen, versican, and fibronectin), an EMT/EndMT driver (SLUG), and inflammatory cytokines and chemokines (e.g. IL-1β, CXCL10, and CXCL11). GO analysis revealed an enrichment of inflammation-related terms, such as ‘defense response’ and ‘inflammatory response’ in this cluster (Fig 1E and 1F). As for cluster 3 genes, while siERG caused a greater change in expression level than siFLI1, siFLI1 in combination with siERG dramatically upregulated gene expression more than either one individually (S2A and S2B Fig). These results clearly indicate that FLI1, as well as ERG, have a non-negligible contribution to mesenchymal conversion.

### ERG and FLI1 regulate gene expression in diverse patterns

The heatmap also shows diverse patterns of ups and downs in gene expression across 8 experimental conditions, some of which are obscure in the current visualization method (Fig 1E). To make the individual functions of ERG and FLI1 more clearly, we performed hierarchical clustering again by using only day 3 datasets, which reflect the direct responses of ERG/FLI1 knockdown. Consequently, we detected 8 major regulation patterns (I–VIII) and other minor patterns (S3 Fig). The list of genes in each pattern is also shown (S1 Table). Note that Z-scores do not coincide with the violin plots in S2A Fig, and each pattern does not necessarily correspond to a certain cluster in Fig 1E, since the expression values are re-normalized within day 3 datasets and newly clustered without day 7 datasets. As for gene sets in which ERG and FLI1 cooperatively represses expression (I–III), ERG predominantly represses expression in the majority of cases (I), while FLI1 can also have a predominant role in repressing specific genes (II). ERG and FLI1 almost redundantly repress certain genes (III). As for gene sets in which ERG individually represses expression (IV and V), an additional knockdown of FLI1 partially (IV) or completely (V) counteracts increased expression by ERG knockdown. This implies that FLI1 (V), along with other TFs (IV), promotes the expression of specific gene sets in the absence of ERG. As for gene sets in which ERG and FLI1 cooperatively promote expression (VII and VIII), again, ERG predominantly promotes expression in the majority of cases (VII) and FLI1 can also have a predominant role in repressing specific genes (VIII). Interestingly, ERG and FLI1 have opposing roles in regulating VI genes; ERG promotes and FLI1 represses expression.

### ERG- and FLI1-binding genomic regions are identified by ChIP-seq analysis

**Fig 2:**
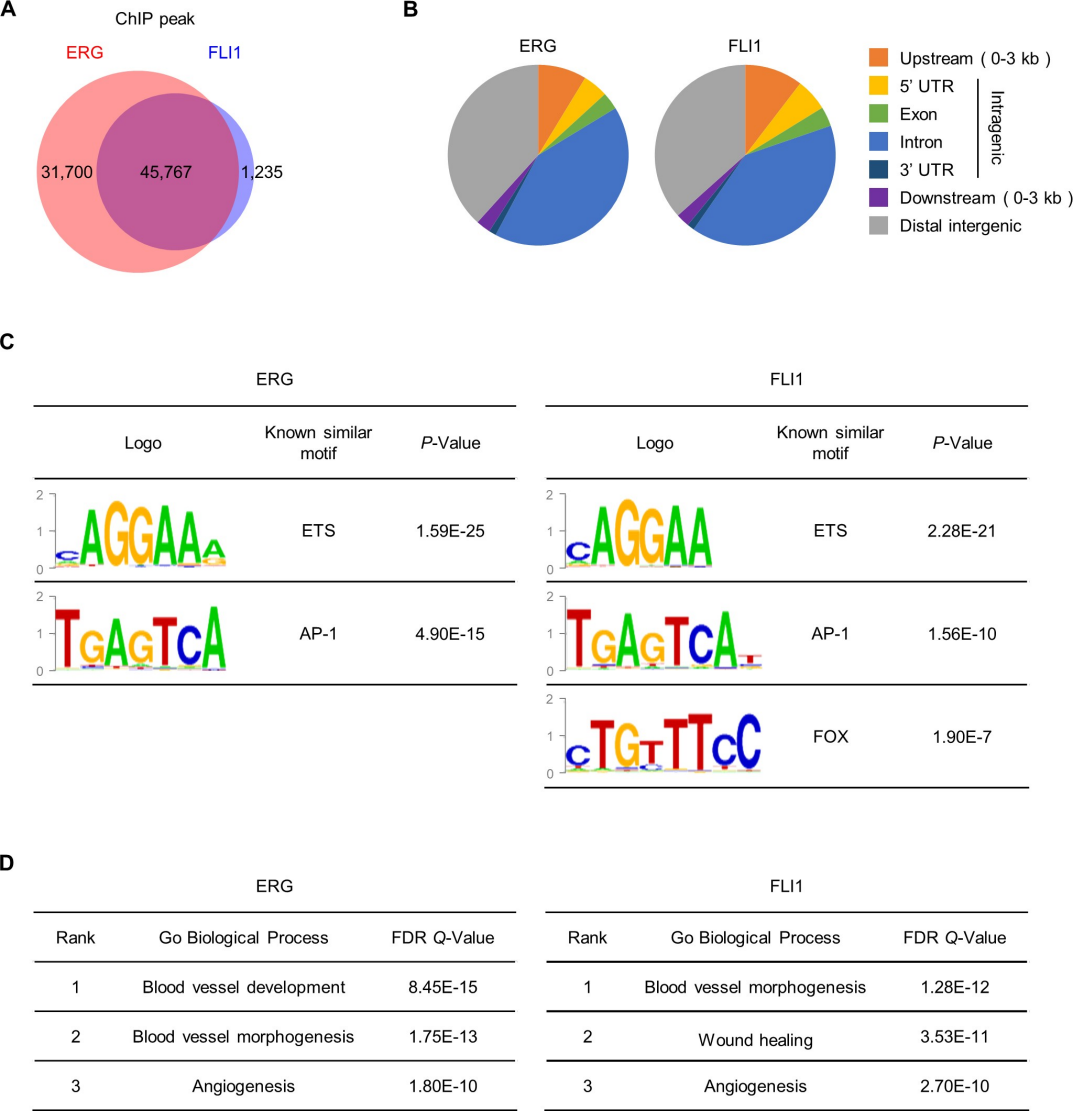
ChIP-seq analysis of ERG and FLI1 in HUVECs. (A) Venn diagram showing the overlap between the ChIP-seq peaks of ERG and FLI1. (B) Peak distributions of ERG and FLI1 in the indicated genomic regions. (C) Motif analysis of ERG and FLI1 in HUVECs. (D) Gene ontology analysis of ERG- and FLI1-binding genes. Genes are selected by assigning the top 2000 peaks to the nearest TSS within 1 Mb. See also S4 Fig.

Next, we evaluated the mechanism of EndMT induction via downregulation of *ERG* and *FLI1* expression by analyzing the functions of these TFs in ECs. We screened the genome-wide binding regions of ERG and FLI1 in HUVECs by ChIP-seq analysis. Prior to performing the ChIP assay, specificities were evaluated by immunoblot analysis to examine the possible cross-reactivity of anti-ERG and anti-FLI1 antibodies given the high structural similarity of these TFs (S4A Fig). Although the commercially available antibodies tested showed some cross-reactivity, antibodies that dominantly detected specific targets were used for the ChIP assay. ChIP-seq revealed 77,467 and 47,002 peaks in ERG and FLI1, respectively (Fig 2A). The peaks of ERG and FLI1 highly overlapped with each other, reflecting the structural similarity of these TFs (Fig 2A). Reproducibility of the ChIP-seq was confirmed by the similarity between two biological replicates as shown in S4B Fig. The peaks of ERG and FLI1 were distributed as shown in Fig 2B and S4C Fig. Motif analysis of ERG and FLI1 showed that the ETS- and AP-1-binding motifs are ranked first and second, respectively (Fig 2C). This is consistent with several reports illustrating the coordinating activity and physical binding of ERG/FLI1 and AP-1 [29, 30]. GO analysis showed that the peaks of ERG and FLI1 are highly enriched in the proximal region of genes associated with vascular function, such as ‘blood vessel morphogenesis’ and ‘angiogenesis’, indicating that these proteins are essential TFs in ECs, as expected (Fig 2D).

### ERG and FLI1 directly promote the expression of a broad range of EC-specific genes

**Fig 3:**
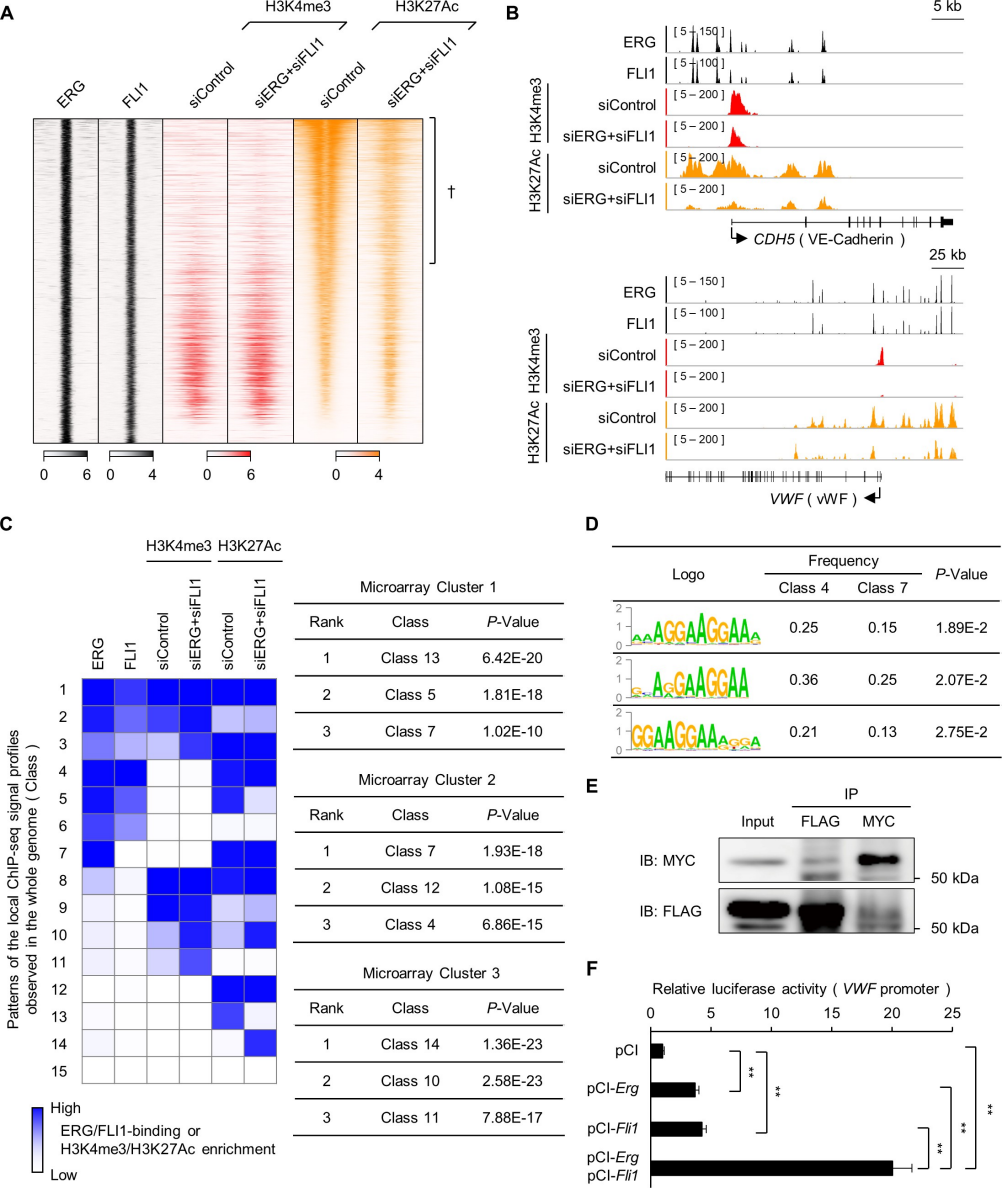
Epigenetic regulation of EndMT mediated by loss of ERG/FLI1. (A) Heatmap showing histone modifications around ERG/FLI1-binding regions. The regions where the H3K27Ac level is significantly decreased after siERG+siFLI1 treatment are marked with a dagger (†). (B) ChIP-seq profiles of ERG, FLI1, and the indicated histone modifications (siControl vs siERG+siFLI1) in HUVECs. Representative EC-specific gene loci, *CDH5* and *VWF*, are shown. (C) Combined analysis of epigenetic (ChIP-seq) and gene expression (microarray) changes. The heatmap (left panel) shows patterns (classes) of local (i.e. 200-bp window) ChIP-seq signal profiles observed over the whole genomic region, determined by the chromHMM program. The right panel shows the top 3 classes highly observed in regulatory regions (i.e. regions within 50 kbp around TSS) of the genes in each microarray cluster. (D) Frequency of the indicated motifs in classes 4 and 7 in Fig 3C. The enrichment of each motif in class 4 compared to class 7 was statistically evaluated and shown as *p*-values. (E) Co-immunoprecipitation assay between ERG and FLI1. Cos-7 cells were transfected with Myc-tagged *Erg* and Flag-tagged *Fli1*, and whole cell lysates were immunoprecipitated with anti-Myc (9E10; mouse monoclonal antibody) or anti-Flag (Flag M2; mouse monoclonal antibody), then subjected to immunoblot analysis with anti-Myc-Tag (71D10; rabbit monoclonal antibody) or anti-Flag (D6W5B; rabbit monoclonal antibody). (F) Luciferase reporter assay for *VWF* gene promoter. Data are represented as mean ± SEM (n = 6). **P < 0.01 by Student’s t-test. See also S5 and S6 Figs.

To further dissect the functions of ERG and FLI1 in ECs, we obtained additional ChIP-seq data for H3K4me3 and H3K27Ac, which are major histone modifications that mark promoters and/or enhancers, in HUVECs treated with siControl and siERG+siFLI1. Reproducibility of the ChIP-seq data was confirmed using two biological replicates (S5A Fig). From a macroscopic perspective, the peaks commonly bound by ERG and FLI1 highly overlapped with H3K27Ac, indicating transcriptionally active regulatory regions (Fig 3A). Additionally, this H3K27Ac was significantly lost by siERG+siFLI1 treatment (Fig 3A). In contrast, the regions bound by ERG/FLI1 and H3K27me3, which marks the transcriptionally repressive state, were mutually exclusive (S5B and S5C Fig). These results indicate that ERG and FLI1 bind genomic regions permissive to TF binding and are associated with gene activation rather than repression. Because the peaks of ERG and FLI1 were enriched in the proximal regions of genes associated with EC function (Fig 2D), we predicted that these TFs may activate the transcription of a wide range of EC-specific genes. Indeed, ChIP-seq data showed that ERG and FLI1 bound to the upstream regions of various EC-specific genes (Fig 3B and S6A Fig). Additionally, these peaks highly overlapped with the H3K27Ac peaks, which were lost with siERG+siFLI1 treatment (Fig 3B and S6A Fig); these results are consistent with the dagger-marked regions in Fig 3A. Loss of H3K27Ac was accompanied by decreased gene expression (S2B Fig). These data clearly showed that ERG and FLI1 directly bind the enhancer/promoter regions of various EC-specific genes and promote their transcription. This accounts for one side of the mechanism of EndMT induction; downregulation of ERG and FLI1 expression leads to a significant decrease in EC-specific gene expression, and thus to the loss of endothelial properties.

### ERG and FLI1 indirectly repress mesenchymal-related genes by epigenetic rearrangement

In addition to changes in histone modification in the regulatory regions of EC-specific genes, we also observed that H3K4me3 and/or H3K27Ac marks increased in the upstream regions of mesenchymal-related genes accompanied by increased gene expression after knockdown of *ERG* and *FLI1* in HUVECs (S2B and S6B Figs). Interestingly, these regions were not necessarily bound by ERG and FLI1. This observation encouraged us to perform a comprehensive analysis on ChIP-seq and gene expression microarray data. Fig 3C shows the local profiles of ChIP-seq signals observed in the whole genomic region which are classified into 15 classes (left panel), and classes which frequently appear in the regulatory regions of the genes in each microarray cluster (Fig 1E) (right panel). For example, genomic regions grouped into class 5 are commonly bound by ERG and FLI1. These regions are marked with H3K27Ac, which are lost upon siERG+siFLI1 treatment. This class is strongly correlated with cluster 1 in the gene expression microarray (*P* = 1.81E-18), confirming that ERG and FLI1 directly regulate the expression of cluster 1 genes which are highly associated with EC-specific genes (Fig 1F). Fig 3C also illustrates that EndMT mediated by ERG/FLI1 loss induces genome-wide changes of histone modifications, even in regions not bound by ERG and FLI1. Particularly, class 14 (*P* = 1.36E-23), 10 (*P* = 2.58E-23), and 11 (*P* = 7.88E-17), which are highly correlated with the regulatory regions of cluster 3 genes, shows very low or no ERG/FLI1 binding, while levels of H3K4me3, H3K27Ac, or both, are increased after siERG+siFLI1 treatment. Taken together, ERG and FLI1 prevent ECs from EndMT by directly promoting EC-specific genes and indirectly repressing EndMT-promoting genes via epigenetic regulation.

### ERG and FLI1 cooperatively activate a cluster of EC-specific genes

Interestingly, a detailed motif analysis on Fig 3C showed that the GGAA repeat sequence was more highly enriched in the ERG^+^FLI1^+^ enhancer (class 4 and 5) than in the ERG^+^FLI1^-^ enhancer (class 7) (Fig 3D). These results suggest that ERG and FLI1 may interact with each other and cooperatively activate certain EC-specific genes. Therefore, we investigated whether ERG and FLI1 have physical contact by performing a co-immunoprecipitation assay for Myc-tagged ERG and Flag-tagged FLI1. As shown in Fig 3E, when precipitated using anti-Myc antibody, co-immunoprecipitated FLI1 was detected and *vice versa*. Furthermore, ERG and FLI1 synergistically drive the activity of *VWF* promoter, an EC-specific gene (Fig 3F). These results indicate that ERG and FLI1 form a complex, and cooperatively regulate the expression of a set of EC-specific genes.

### MicroRNA-126 protects ECs from EndMT downstream of ERG and FLI1

To identify the critical downstream target of ERG/FLI1 with EndMT-inhibiting function, we globally screened genes bound and transcriptionally activated by ERG and FLI1 using ChIP-seq and microarray data. Genes that meet the following three criteria in ChIP-seq were listed: 1) the upstream region has ERG/FLI1-binding peaks, 2) ERG/FLI1 peaks overlap with H3K27Ac peaks, and 3) the H3K27Ac peaks are reduced by siERG+siFLI1. Moreover, genes that meet the following two criteria in microarrays were listed: 1) siERG+siFLI1 reduces expression by >70%, and 2) siERG+siFLI1 reduces expression more than siERG or siFLI1 alone. Finally, 293 candidate genes were commonly listed (S7A Fig and S2 Table). Through all screenings, *SMAD1* was a possible candidate. During manuscript preparation, it was reported that ERG controls the TGFβ/SMAD signaling pathway to block EndMT by promoting SMAD1 expression and inhibiting DNA binding of SMAD3 in, for example, *CNN1* and *TGFB2* promoters [26]. Consistent with this finding, our ChIP-seq and microarray data clearly showed that ERG and FLI1 bound the *SMAD1* promoter, and combined knockdown of ERG/FLI1 reduced SMAD1 expression (S8A Fig). Moreover, ERG and FLI1 bound promoter regions of *CNN1* and *TGFB2*, and combined knockdown of ERG/FLI1 increased their expression (S8B Fig). Taken together, these data suggest that FLI1 as well as ERG can modify the SMAD pathway to protect ECs from EndMT.

**Fig 4:**
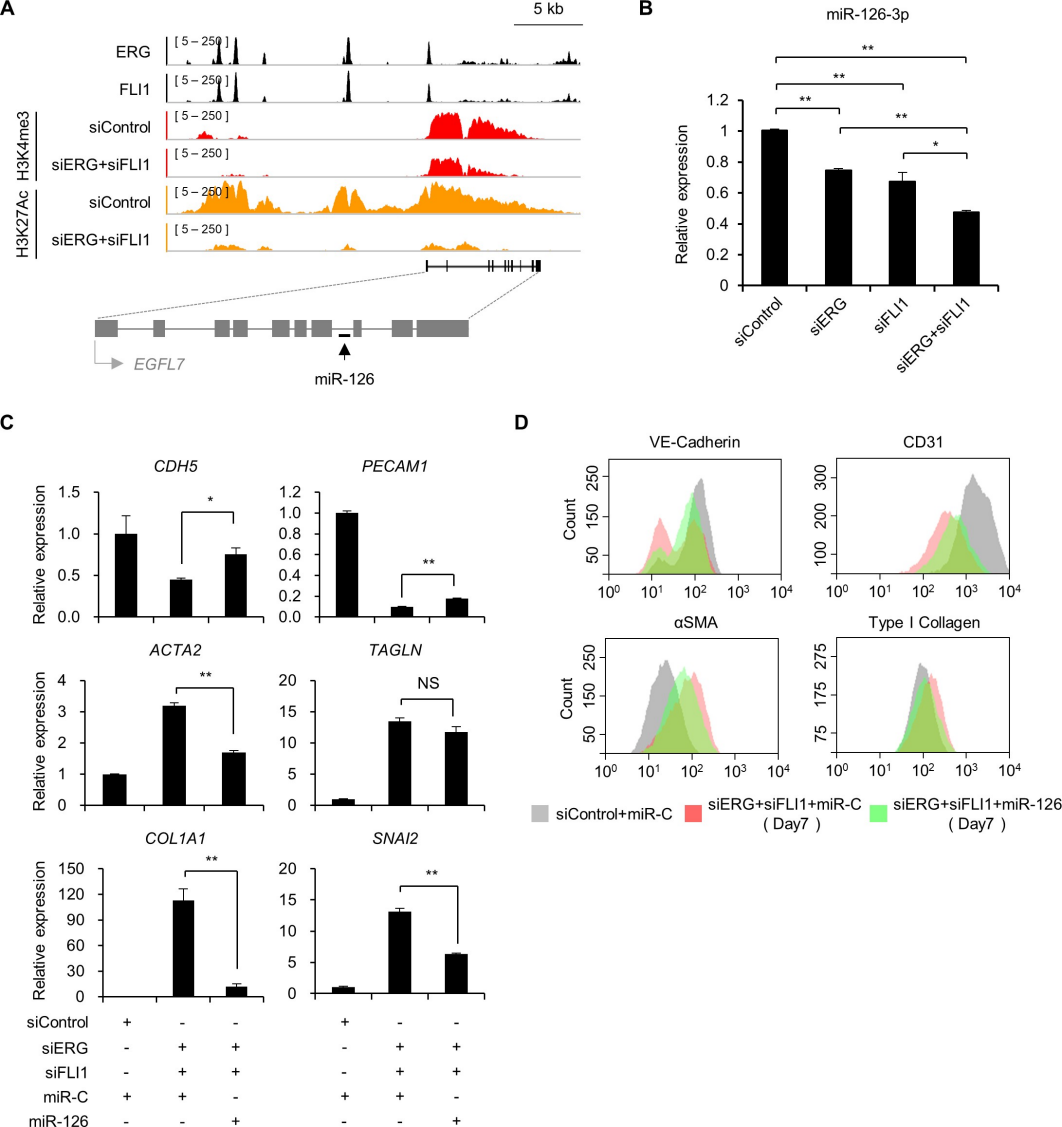
microRNA-126 protects ECs from EndMT downstream of ERG and FLI1. (A) ChIP-seq profiles of ERG, FLI1, and indicated histone modifications (siControl vs siERG+siFLI1) around *EGFL7*/microRNA-126 loci in HUVECs. (B) Relative expression of microRNA-126-3p quantified by qPCR in HUVECs treated with siERG, siFLI1, or both for 3 days. Data are represented as mean ± SEM (n = 3). *P < 0.05; **P < 0.01 by Student’s *t*-test. (C) Relative expression of endothelial/mesenchymal markers quantified by qPCR upon restoration of miR-126. HUVECs were treated with siControl+miR-Control (miR-C), siERG+siFLI1+miR-Control or siERG+siFLI1+miR-126 mimic for 3 days. Data are represented as mean ± SEM (n = 3). *P < 0.05; **P < 0.01 by Student’s *t*-test. NS, not significant. (D) Flow cytometry analysis of endothelial/mesenchymal marker expression. HUVECs were treated with siControl+miR-Control, siERG+siFLI1+miR-Control or siERG+siFLI1+miR-126 mimic for 7 days. See also S10 Fig.

Subsequently, to identify a new molecule, we collected information on microRNA, as microRNAs are a well-recognized regulator of EMT [31]. Among the microRNAs related to EMT/EndMT, we searched for the downstream target of ERG/FLI1 (S7B Fig). As a result, our ChIP-seq data indicated that microRNA-126 (miR-126) is the most promising direct target because ERG and FLI1 bind the enhancer/promoter regions of miR-126, and these regions overlap with H3K27Ac, which was significantly lost after siERG+siFLI1 treatment (Fig 4A). Moreover, miR-126 expression was significantly decreased by combined knockdown of *ERG* and *FLI1*, indicating that these TFs directly promote the expression of miR-126 (Fig 4B). In contrast, we failed to detect ERG/FLI1 peaks and/or significant changes in histone modification enrichment in the other EMT/EndMT-related microRNAs such as the let-7 family and the miR-200 family after knockdown of *ERG* and *FLI1* (S9 Fig).

MiR-126 is an EC-specific microRNA located in intron 7 of *EGFL7*, which is also an EC-specific gene. Interestingly, MirDIP microRNA target prediction database [32] indicated that mesenchymal-related genes involving *TAGLN*, *COL1A1*, and *SNAI2* are potential targets of miR-126 (S3 Table). Moreover, a recent study showed that miR-126 blocks TGFβ-induced EndMT by targeting *PIK3R2* mRNA [33, 34]. Thus, we investigated whether restoration of miR-126 counteracts endothelial/mesenchymal marker expression changes through downregulation of ERG and FLI1 expression. As shown in Fig 4C and 4D, transfection with a miR-126 mimic partially counteracted decreased expression of *CDH5* and *PECAM1*. A miR-126 mimic also counteracted increased expression of *ACTA2*, *COL1A1*, and *SNAI2*, but not *TAGLN*. In contrast, a miR-126 inhibitor induced partial EndMT in HUVECs, indicated by the downregulation of *CDH5* expression and the upregulation of *TAGLN*, *COL1A1*, and *SNAI2* expression (S10 Fig). Taken together, these data suggest that EndMT mediated by the loss of ERG/FLI1 is at least in part based on the reduced expression of miR-126 under the direct control of these TFs.

### ERG and FLI1 expression are downregulated in intratumoral ECs

**Fig 5:**
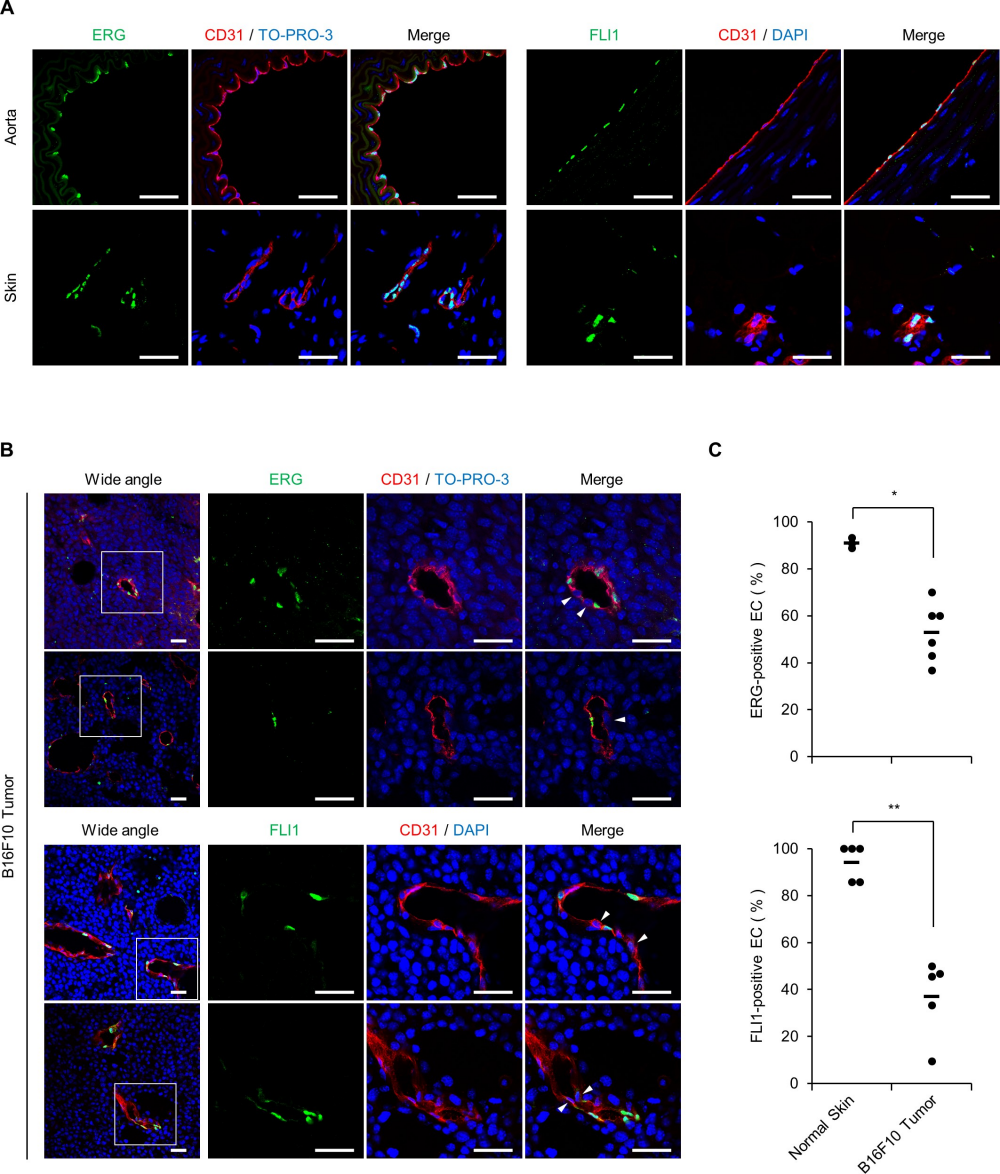
ERG and FLI1 expression is downregulated in intratumoral ECs. (A) Representative immunofluorescent staining of normal ECs in aorta and skin. Endothelial marker CD31, red; ERG and FLI1, green; nuclei, blue. (B) Representative immunofluorescent staining of ECs in B16F10 tumor tissues. Endothelial marker CD31, red; ERG and FLI1, green; nuclei, blue. The boxed region in the left image is magnified in the right three images. Arrows indicate ERG- or FLI1-negative ECs. (C) Quantification of ERG-positive or FLI1-positive CD31^+^ ECs. Each plot shows positive ratios in a single image and the horizontal line shows the average. *P < 0.05; **P < 0.01 by Mann-Whitney *U* test. Immunofluorescent staining was reproduced in at least 5 independent mice. Scale bar, 250 μm. See also S11 Fig.

Because EndMT is known to be induced in the tumor microenvironment [5], we investigated the expression of ERG and FLI1 in ECs in tumor tissues by immunofluorescent staining. Note that the anti-ERG antibody used for immunostaining differed from that used for ChIP-seq and shows cross-reactivity with FLI1 as described in the manufacturer’s datasheet (S4A Fig). First, we observed the expression of ERG and FLI1 in normal aorta and skin ECs. Consistent with the fact that ERG has been widely recognized as an EC marker in immunostaining assays and previous reports showing strong detection of FLI1 in ECs [35], strong and homogenous expression of these TFs were observed in CD31^+^ ECs (Fig 5A). In contrast, within tumors formed after subcutaneous injection of B16F10 melanoma cells, some ECs showed reduced expression of ERG and FLI1 (Fig 5B and 5C). Similar results were observed in tumor tissues formed by E0771 cells (intra-fat pad) and 3LL cells (subcutaneous) (S11A and S11B Fig). These data suggest that EndMT mediated by ERG/FLI1 loss is induced in the tumor microenvironment *in vivo*.

**Fig 6:**
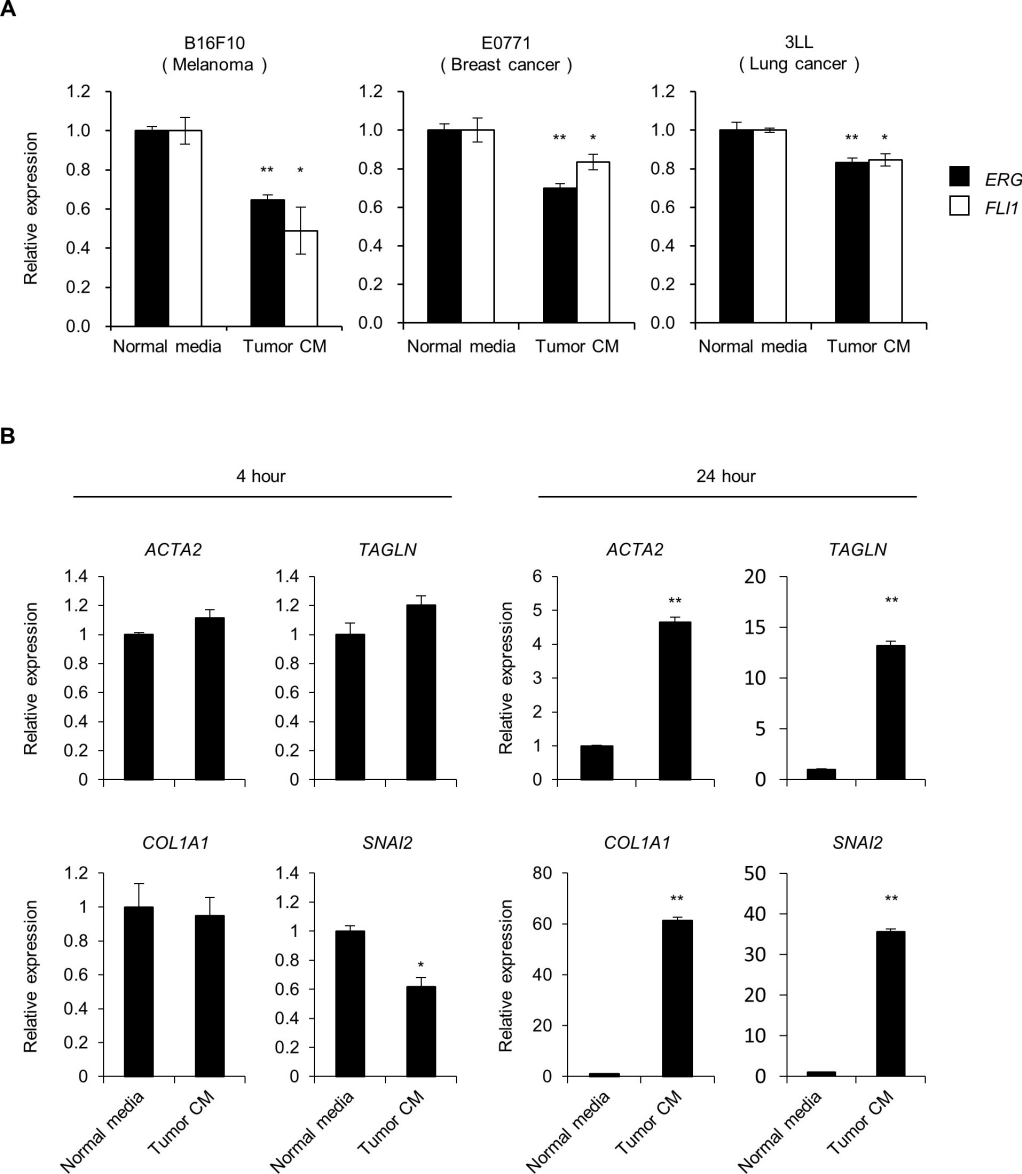
*ERG* and *FLI1* expression is downregulated by soluble factors enriched in the tumor microenvironment. (A) Relative expression of *ERG* and *FLI1* quantified by qPCR in HUVECs treated with normal starvation media (Normal media) or tumor tissue-conditioned media (Tumor CM) for 4 hours. Tumor CM was collected from whole cells obtained by digesting tumor tissues with collagenase. Tumor tissues formed by B16F10 cells, E0771 cells, and 3LL cells were used. Data are represented as mean ± SEM (n = 3–8). *P < 0.05; **P < 0.01 by Student’s *t*-test. (B) Relative expression of mesenchymal markers quantified by qPCR in HUVECs treated with normal starvation media (Normal media) or B16F10 tumor tissue-conditioned media (Tumor CM) for 4 or 24 hours. Data are represented as mean ± SEM (n = 3). *P < 0.05; **P < 0.01 by Student’s t-test. See also S12 Fig.

We then evaluated the cause of downregulation of ERG and FLI1 expression in ECs within tumors. Previous studies reported that the extracellular environment including soluble factors represented by inflammatory cytokines, hypoxia, and high glucose trigger EndMT under pathological conditions [4, 36]. Among these, we investigated whether soluble factors and hypoxia reduce the expression of ERG and FLI1, as aberrant soluble factor profiles and hypoxia are key characteristics of the tumor microenvironment. First, to examine the effect of soluble factors, we evaluated expression changes of *ERG* and *FLI1* in HUVECs treated with culture media conditioned with intratumoral whole cell populations. As a result, *ERG* and *FLI1* expression was significantly decreased after a 4-hour conditioned media treatment by all three implanted tumors investigated, indicating that expression of these TFs is at least partially downregulated by soluble factors enriched in the tumor microenvironment (Fig 6A). In addition, the 24-hour treatment with B16F10 tumor tissue-conditioned media induced the expression of mesenchymal markers in HUVECs while the 4-hour treatment did not (Fig 6B). We also found that various inflammatory cytokines, particularly TNFα, IL-1β and IFNγ downregulated the expression of *ERG* and/or *FLI1* in HUVECs (S12A Fig). In contrast, cobalt chloride, which is known to induce hypoxia by activating HIF-1, did not downregulate the expression of *ERG* or *FLI1* (S12B Fig). These results suggest that soluble factors play a key role in promoting EndMT by repressing ERG/FLI1 expression in the tumor environment.

### EndMT mediated by loss of ERG/FLI1 in clinical cancer patients

To assess the relevance between EndMT mediated by loss of ERG/FLI1 and cancer progression, we analyzed Kaplan-Meier plots obtained from the PrognoScan database (http://www.abren.net/PrognoScan/). In consideration of the technical limitations of PrognoScan-based Kaplan-Meier plots, which were constructed based on the transcriptome of whole tumor tissues, we set two criteria as follows: (1) ERG is used as a prognostic marker because its expression is highly limited to ECs, while FLI1 is expressed in other cell populations such as myeloid cells [13]; (2) the probe set 213541_s_at is used because it is validated in the current study. Under these conditions, we found that lower expression of *ERG* was significantly related to poor prognosis in melanoma (overall survival), breast cancer (disease-specific survival), and lung cancer (overall survival) (S13A and S13B Fig). These data support the idea that EndMT mediated by loss of ERG/FLI1 promotes tumor progression.

## Discussion

**Fig 7:**
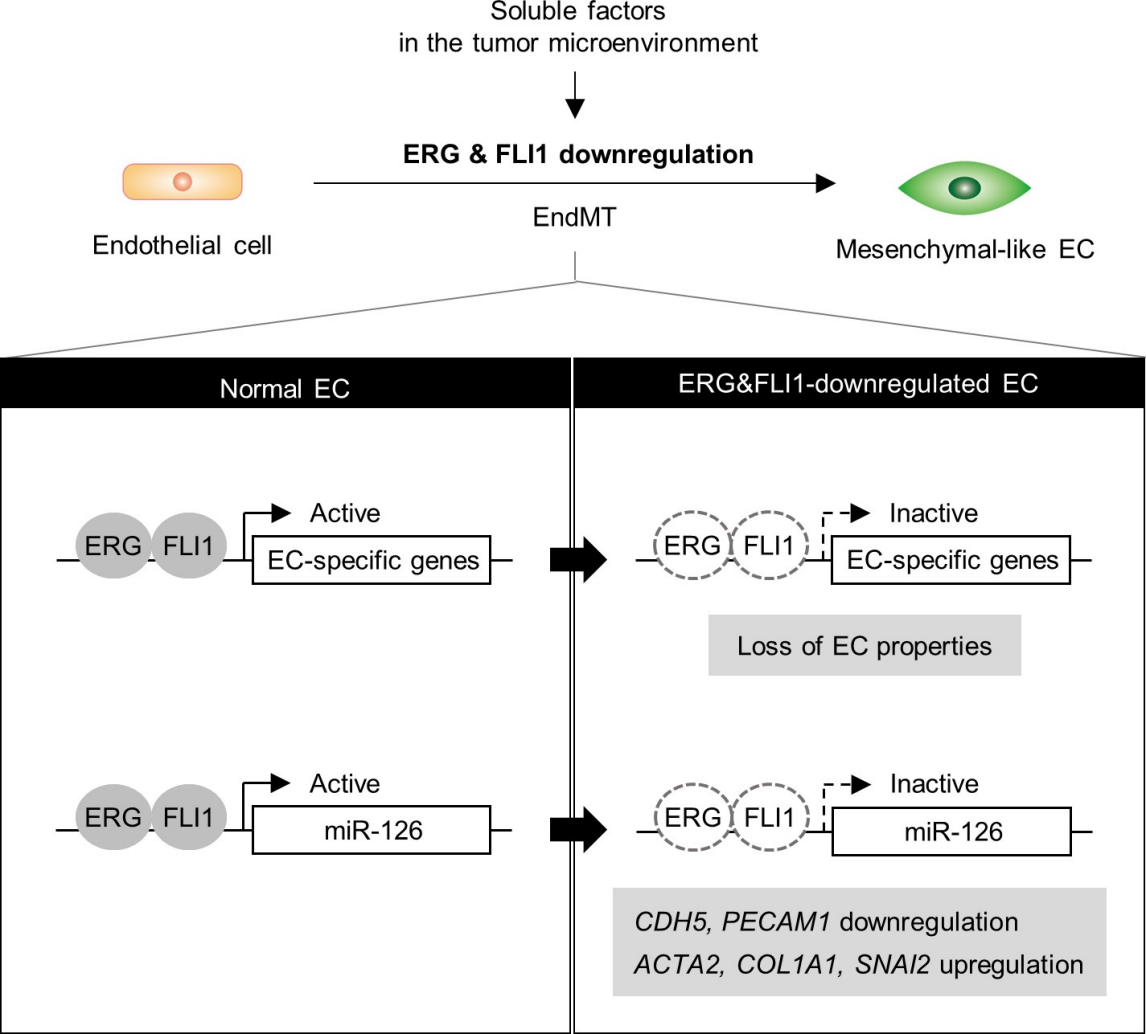
Loss of *ERG* and *FLI1* triggers EndMT via downregulation of EC-specific genes and miR-126. ERG and FLI1 activate the transcription of EC-specific genes and miR-126, and maintain EC properties. In the tumor microenvironment, expression of *ERG* and *FLI1* is downregulated in ECs by soluble factors derived from tumor milieu, which in turn facilitates EndMT.

In the current study, we found that the expression of ERG and FLI1 in tumor ECs is downregulated because of soluble factors enriched in the tumor microenvironment. Reduced expression of ERG and FLI1 resulted in the loss of a broad range of EC-specific genes under the direct control of these TFs, leading to the loss of endothelial characteristics. Further, among the EC-specific genes transcriptionally activated by ERG and FLI1, we found that miR-126 partially blocks EndMT, the loss of which triggers EndMT (Fig 7). Additionally, we gained genome-wide insight into the functions of ERG and FLI1, which have been recently recognized as essential TFs in EC differentiation and function [37].

ERG directly regulates several EC-specific genes to maintain EC function [19–26]. Additionally, reduced expression of ERG leads to upregulation of *CXCL8*, *ICAM1*, and *VCAM1*, which are representative genes in inflammatory ECs, suggesting that ERG is a “gatekeeper” against inflammatory phenotypes [27, 28, 38]. In contrast to ERG, the function of FLI1 is less-characterized in ECs; its ablation results in upregulation of *CTSL* and *CXCL6* and downregulation of *CXCL5* and *CCN1* in the context of systemic sclerosis [39–42]. Our ChIP-seq and microarray analysis found that ERG and FLI1 bind enhancer/promoter regions and directly regulate various EC-specific genes, some of which are first characterized by our genome-wide study. Moreover, our study indicated the synergistic role of ERG and FLI1; they can interact physically (Fig 3E), and synergistically transactivate *VWF* promoter activity (Fig 3F). Notably, ERG also interacts with other molecules such as NFκB and SMAD3 to regulate gene transcription [26, 28, 38]. Thus, the molecular interaction networks of ERG and FLI1 need to be further analyzed to clarify the whole mechanism of mesenchymal transition.

ERG and FLI1, individually or in combination, have diverse modes of gene regulation (S3 Fig). In support of the idea that ERG and FLI1 have a physical interaction, some regulation patterns indicate that these TFs mutually support each other’s function (I, II, III, VII, and VIII in S3 Fig). In this case, ERG usually has a predominant role in regulating gene expression (I, VII). Moreover, ERG and FLI1 may change a function depending on whether they form a complex or not; FLI1 can drive a set of genes only in the absence of ERG (IV and V in S3 Fig). Interestingly, pattern V gene sets include EC inflammatory genes (*ICAM1* and *CXCL8*), raising the hypothesis that FLI1 triggers an inflammatory state in response to the loss of ERG as an “emergency signal”. In contrast, ERG and FLI1 occasionally have opposing roles; ERG promotes, but FLI1 represses the expression of pattern VI gene sets. This raises another possible hypothesis that FLI1 can fine-tune ERG-driven upregulated expression in regulating a subset of genes through direct contact. In contrast to the combinatorial effect of siERG and siFLI1, detailed results induced by siERG or siFLI1 alone are inconsistent between figures and with the literature. For example, *ACTA2* is downregulated by siERG in S1B Fig, but upregulated in Fig 1C, S2B Fig, and the literature [26]. In addition, *TAGLN* is upregulated further by siERG alone than by siERG+siFLI1 in Fig 1C, which is inconsistent with the other figures. These inconsistencies are possibly due to the difference in experimental techniques (qPCR and microarray), materials (siRNAs), and primary HUVEC lots and passage numbers. Further specialized analysis will be needed to precisely determine the individual role of ERG and FLI1 in regulating a certain gene.

Transcriptional activities of ERG and FLI1 can also be regulated in a post-translational manner. For example, ERG is activated by phosphorylation at serine 96, 215, and 276 in ECs [43]. In contrast, the transcriptional activity of FLI1 is disrupted by phosphorylation at threonine 312 and subsequent acetylation at lysine 380 via the non-canonical TGFβ signaling pathway [44]. These data indicate that EndMT can be triggered by attenuated functions of ERG and FLI1 as well as by reduced mRNA expression of these TFs.

Through genome-wide target screenings, we focused on miR-126 as a major EndMT-inhibiting factor under the direct control of ERG and FLI1 (Fig 4). MiR-126 is an EC-specific microRNA that maintains EC function by reinforcing VEGFR signaling [45, 46]. In support of our finding, miR-126 knockout mice show phenotypes similar to *Erg* and *Fli1* knockout mice, suggesting that miR-126 is a pivotal downstream molecule of ERG and FLI1 [14–17, 45]. It has been previously reported that miR-126 is transcriptionally activated by ETS1 and ETS2 [45, 47], but neither ERG nor FLI1 can activate a proximal promoter of miR-126 [47]. In the current study, we found that these TFs bind not only to a proximal promoter but also to distal regulatory regions of miR-126. Coupled with histone modification changes by ERG/FLI1 knockdown, we conclude that ERG/FLI1 can promote miR-126 expression. MiR-126 has been shown to target *PIK3R2* mRNA, which codes for a negative regulator of the PI3K/Akt signaling pathway, to protect ECs from EndMT in the context of TGFβ1-induced EndMT. Loss of miR-126 results in diminished activation of PI3K/Akt signaling and subsequent nuclear translocation of FOXO3a, which cooperates with SMAD3/4 to activate EndMT program genes [33, 34]. We therefore assessed the contribution of the PIK3R2-PI3K/Akt-FOXO3a axis to EndMT mediated by the loss of ERG/FLI1. However, we failed to find significant upregulation of *PIK3R2* in HUVECs treated with siERG+siFLI1, despite clear downregulation of miR-126 (S14A Fig). This observation raises a possibility that another downstream pathway works in EndMT mediated by ERG/FLI1 loss. *SNAI2* and *TWIST2*, well-characterized EMT/EndMT regulators, are significantly upregulated upon combined knockdown of *ERG* and *FLI1*, but *SNAI2* or *TWIST2* knockdown could not counteract the endothelial/mesenchymal marker expression changes through the downregulation of ERG and FLI1 expression (S14B and S14C Fig). Although ERG directly binds and transcriptionally activates *SNAI2* in the context of endocardial-to-mesenchymal transition during heart development [14], we observed upregulation of *SNAI2* expression after siERG treatment, probably due to the difference between ECs and endocardial cells, or postnatal and embryonic stages. Another well-known EndMT inducer, TGFβ2, was upregulated by suppression of ERG and FLI1 in HUVECs (S8 Fig). Thus, TGFβ2 and the downstream SMAD signaling pathway may enhance mesenchymal transition in an autocrine manner, which needs to be further investigated.

Previous studies showed that ERG or FLI1 expression was downregulated in atherosclerosis, systemic sclerosis, pulmonary arterial hypertension, and liver fibrosis [26, 38, 48, 49]. In these reports, ablation of ERG and/or FLI1 in ECs resulted in upregulation of cytokine and chemokine expression and showed inflammatory phenotypes, consistent with our study (Fig 3E and 3F). Here, we additionally show that ERG and FLI1 expression is also downregulated in cancer pathology. Given that inflammation is a well-known pro-tumor factor [50], ECs that have undergone EndMT mediated by ERG/FLI1 loss may promote tumor progression in an inflammation-dependent manner. It has been shown that TNFα and IL-1β downregulate *ERG* expression, and IFNγ downregulates *FLI1* expression [26, 27, 38, 51]. In the current study, we found that soluble factors enriched in tumor tissues are responsible for reduced expression of *ERG* and *FLI1* (Fig 6). In the tumor microenvironment, ERG and FLI1 can be downregulated via the mixture of cytokines from many sources such as infiltrating immune cells (e.g. neutrophils, macrophages, and NK cells), ECs, CAFs, and tumor parenchyma. In addition, TNFα, IL-1β, IFNγ, and TGFβ have been shown to induce EndMT [36, 52, 53]. Some studies reported that the combination of inflammatory cytokines synergistically induce EndMT, which may be attributed to the synergistic downregulation of ERG and FLI1 expression [52, 53]. Taken together, these data suggest that multiple cytokine dynamics rather than a single cytokine-mediated signaling would lead to EndMT via ERG and FLI1 reduction in the tumor microenvironment.

In conclusion, we identify ERG and FLI1 as critical regulators of EndMT in ECs. ERG and FLI1, individually or in combination, directly induce EC-specific genes and indirectly repress mesenchymal genes by epigenetic regulation. Importantly, ERG and FLI1 cooperatively regulate a set of EC-specific genes, indicating that both TFs are important for EC function. Our work delineates the role of ERG and FLI1 in ECs, and suggests that maintaining the expression of these TFs may be possible therapeutic options for various EndMT-related diseases including cancer.

## Materials and methods

### Ethics statement

The Animal Care and Use Committee of the University of Tokyo and The Animal Care and Use Committee of Kumamoto University School of Medicine approved the study (A29-070R2). The work was conducted according to guidelines issued by the Center for Animal Resources and Development of Japan. The guideline totally following the international animal research rule with 3R (Replacement, Reduction and Refinement).

### Cell culture

Human umbilical vein endothelial cells (HUVECs) were purchased from Lonza (Basel, Switzerland). HUVECs were cultured in EGM-2 (CC-3162, Lonza) supplemented with 5% fetal bovine serum (FBS) in a humidified atmosphere of 5% CO_2_ at 37°C. B16F10 cells, E0771 cells, and 3LL-luc cells (3LL cells which constitutively expresses luciferase gene) were cultured in DMEM (D5796; Sigma, St. Louis, MO, USA) supplemented with 10% FBS. 3LL-luc and E0771 were kind gifts from Dr. Yoshihiro Hayakawa (Toyama University, Japan) and Dr. Robin Anderson (Peter MacCallum Cancer Centre, Australia), respectively.

### siRNA, miRNA, and miRNA inhibitor treatment

HUVECs were treated with siRNAs (4 nM), miRNA mimics (30 nM), or miRNA inhibitor (100nM) using RNAiMAX Transfection Reagent (13778075, Thermo Fisher Scientific, Waltham, MA, USA) following the manufacturer’s instructions. In the control experiment, comparable concentrations of control siRNAs/miRNAs/miRNA inhibitor produced by the same manufacturer were transfected. A list of siRNAs and miRNAs is shown in S4 Table.

### Quantitative RT-PCR

#### mRNA quantification

Total RNA was extracted from cells using TRIReagent (TR118, Molecular Research Center, Cincinnati, OH, USA) following the manufacturer’s instructions. Next, 0.5 μg of total RNA was reverse-transcribed with PrimeScript RT Master MIX (RR036, Takara Bio, Shiga, Japan) following the manufacturer’s instructions. Quantitative PCR was performed in triplicate using THUNDERBIRD SYBR qPCR Mix (QPS-201, Toyobo, Osaka, Japan) on Thermal Cycler Dice Real Time System II (TP900, Takara Bio). Relative expression was determined by the standard curve method. *PPIA* mRNA was used as an internal control. A list of primers is described in S5 Table.

#### microRNA quantification

Total RNA was extracted from the cells using an mirVana miRNA Isolation Kit (AM1560, Thermo Fisher Scientific) following the manufacturer’s instructions. Next, 0.25 μg of total RNA was reverse-transcribed with the Taqman MicroRNA Reverse Transcription Kit (4366596, Thermo Fisher Scientific) and TaqMan MicroRNA Assays (Assay ID 002228 and 001093, Thermo Fisher Scientific) following the manufacturer’s instructions. Quantitative PCR was performed in triplicate using THUNDERBIRD Probe qPCR Mix (QPS-101, Toyobo) on an ABI RealTimePCR 7500 Fast (Thermo Fisher Scientific). Relative expression was determined by the ΔΔCt method. *RNU6B* RNA was used as an internal control.

### Immunoblot analysis

HUVECs treated with siRNAs for 3 days were harvested with a cell scraper. Whole cell lysates were prepared using lysis buffer (1% NP-40, 10% glycerol, 137 mM NaCl, 20 mM Tris-HCl, 1.5 mM MgCl_2_, and 1 mM EDTA) containing cOmplete protease inhibitor cocktail (11873580001, Roche, Basel, Switzerland). The protein concentration of the whole cell lysate was quantified with a Pierce BCA Protein Assay Kit (23227, Thermo Fisher Scientific). After preparing samples using Sample Buffer Solution with Reducing Reagent (09499–14, Nacalai, Kyoto, Japan), 50 μg of protein was separated by SDS-PAGE and transferred to PVDF membranes (10600023, GE Healthcare, Little Chalfont, UK). Immunoblots were blocked with 5% skimmed milk/TBST (used also for antibody diluent below) for 1 hour and subsequently incubated with anti-ERG (1:1000; ab136152, Abcam, Cambridge, UK), anti-ERG (1:1000; ab92513, Abcam), anti-FLI1 (1:5000; ab15289, Abcam), and anti-Caspase3 (1:1000; 9662, Cell Signaling Technology, Danvers, MA, USA) overnight at 4°C. After the blots were washed with TBST, they were incubated with HRP-conjugated anti-mouse IgG (1:80,000; A9044, Sigma) or anti-rabbit IgG (1:80,000; A9169, Sigma) overnight at 4°C. After washing with TBST, chemiluminescent signals on the blots were detected using Chemi-Lumi One Super (02230, Nacalai) on an ImageQuant LAS 4000 mini (GE Healthcare). For loading controls, the blots were stripped with WB Stripping Solution (05364–55, Nacalai) and reprobed with an antibody against β-actin (1:2000; A1978, Sigma).

### Flow cytometry

Cells were detached using 0.2% EDTA/PBS. For VE-cadherin and CD31 staining, cells were blocked with 2% bovine serum albumins (BSA) (019–23293, Wako, Osaka, Japan)/PBS for 30 minutes. For αSMA and collagen type I staining, the cells were fixed in 4% paraformaldehyde (09154–85, Nacalai) for 10 minutes at room temperature, and blocked/permeabilized with 2% BSA/0.1% Triton X-100/PBS for 30 minutes. The cells were incubated with primary and subsequently secondary antibodies in 2% BSA/PBS for 1 hour at 4°C. Samples were analyzed with Guava easyCyte (Millipore, Billerica, MA, USA). Antibodies used were Cy3-conjugated anti-αSMA (1:500; C6198, Sigma), PE-conjugated anti-CD31 (1:50; 303105, Biolegend, San Diego, CA, USA), anti-collagen type I (1:80; AB758, Millipore) and Alexa Fluor 647-conjugated anti-goat IgG (1:200; A-21447, Thermo Fisher Scientific), and Alexa Fluor 647-conjugated anti-VE-cadherin (1:50; 561567, BD Biosciences, San Jose, CA, USA).

### Microarray analysis and heatmap

#### Microarray

Total RNA was extracted from cells using TRIReagent following the manufacturer’s protocol. Gene expression microarray was performed using GeneChip Human Genome U133 Plus 2.0 Array (Affymetrix, Santa Clara, CA, USA). Raw signal values were normalized by Affymetrix Microarray Suite 5.0 (MAS5). After exclusion of the probe sets which did not have annotated genes, the remaining probe sets were used for further analysis.

#### Heatmap

Differentially expressed genes were selected according to the following criteria: (1) the probe sets of which signal values were ≥300 in at least one group among 8 groups (siControl vs siERG vs siFLI1 vs siERG+siFLI1, day 3 vs day 7), (2) the probe sets of which coefficients of variation (CV) were ≥0.5. If a single gene has two or more probe sets, the probe set whose sum of signal values in all groups was the largest was selected. Z-score was calculated for the selected genes and clustered using the HOPACH algorithm [54] with default settings.

### Morphological analysis

HUVECs were seeded into 35-mm dishes. After siRNA treatment, images were captured at hourly intervals for 72 hours with BioStudio (Nikon, Tokyo, Japan).

### Tube formation assay

The mixture of 320 μL of Atelo Collagen (IAC-30, Koken, Tokyo, Japan), 40 μL of 10× MEM (1430030, Thermo Fisher Scientific), and 40 μL of 10× neutralization buffer was plated in a 24-well plate and incubated for 1 hour at 37°C. The 10× neutralization buffer contained 0.1 M HEPES (17557–94, Nacalai) and 0.1 M NaHCO_3_. HUVECs treated with siRNAs for 7 days were seeded onto the solidified gel and incubated overnight. After the medium was removed, 250 μL of collagen gel mixture was layered on the cells and incubated for 1 hour at 37°C. Finally, 500 μL of EGM-2 supplemented with 5% FBS and 50 ng/mL VEGF (223–01311, Wako) were added. After 48-hour incubation, tube formation was observed with a bright field microscope.

### ChIP-seq

ChIP-seq was performed as described previously [37, 55]. Cells were fixed with 1% formaldehyde (061–00416, Wako) for 10 minutes or 0.5% formaldehyde for 5 minutes at room temperature, and then added at a concentration of 200 μM of glycine to stop the reaction. Cells were harvested with a cell scraper. Chromatin was sheared to 150–1000 bp with a SONIFIER 250 (Branson, Danbury, CT, USA) or DNA Shearing system S200 (Covaris, Woburn, MA, USA). Immunoprecipitation was performed with anti-ERG (ab136152, Abcam), anti-FLI1 (ab15289, Abcam; sc-356, Santa Cruz Biotechnology, Dallas, TX, USA), anti-H3K4me3 (MABI0304, MBL, Nagoya, Japan), and anti-H3K27Ac (MABI0309, MBL) which were bound to Dynabeads M-280 Sheep Anti-Mouse IgG (11201D, Themo Fisher Scientific), Dynabeads M-280 Sheep Anti-Rabbit IgG (11203D, Themo Fisher Scientific), or Dynabeads Protein G (10004D, Themo Fisher Scientific). After the beads were washed, resuspended in elution buffer (1% SDS, 0.1 M NaHCO_3_) containing 1 mg/mL Pronase (10165921001, Roche) for at least 2 hours at 42°C and subsequently at 65°C overnight. DNA was purified with a QIAquick PCR Purification Kit (28106, QIAGEN, Hilden, Germany) following the manufacturer’s instructions, and quantified with a Qubit 3.0 Fluormeter (Q33216, Thermo Fisher Scientific) and Qubit dsDNA HS Assay Kit (Q32851, Thermo Fisher Scientific). The sequence library was prepared from 2.5 ng DNA with a KAPA Hyper Prep Kit for illumina following the manufacturer’s protocol (KK8502, Kapa Biosystems, Wilmington, MA, USA) and sequenced with a Genome Analyzer IIx (Illumina, San Diego, CA, USA) or HiSeq 2000 (Illumina). Protocols for each antibody are summarized in S6 Table.

### Computational analysis and bioinformatics

#### ChIP-seq data processing

ChIP-seq data were processed as described previously using the reference genome hg19 [37]. Briefly, 36-bp single-end reads were mapped onto the reference genome hg19 using the ELAND program on a CASAVA platform (Illumina). For the HiSeq run, 50-bp single-end reads were trimmed into 36-bp reads before mapping onto the reference genome. Uniquely mapped reads were used for peak calling using MACS in a default setting [56]. ChIP-seq data were visualized on Integrative Genomics Viewer [57].

#### Creating heatmaps and peak distribution analyses for ChIP-seq data

Heatmaps of ChIP-seq data for ERG, FLI1, H3K4me3, and H3K27Ac were created using ngs.plot [58]. ERG/FLI1-binding peaks with top 5000 scores were extracted and drawn in heatmaps. Peak distribution analysis around whole genomic regions was performed using CEAS [59]. The regions within 3-kbp upstream of the transcription start site (TSS) and 3-kbp downstream of the transcription end site (TES) were defined as ‘upstream’ and ‘downstream’ regions of the genes. Peak distribution analysis around TSS and ERG- and/or FLI1-binding peaks was performed using ngs.plot. To obtain ChIP-seq data for H3K27me3 in HUVECs, a bam file was downloaded from ENCODE (Accession No. ENCFF921ASC).

#### *De novo* motif analysis

*De novo* motif analysis was performed with the MODIC motif identification program in the following settings: window size, 8 bp; background genomes, random genomes; enrichment ratio, >2.0-fold enrichment [60]. The motifs were assigned to known similar transcription factor-binding motifs using the STAMP tool [61].

#### GO analysis

For gene clusters in the microarray, DAVID [62] was used for GO analysis. For ChIP-seq peaks of ERG and FLI1, the peaks with top 2000 scores were applied to GREAT [63]. Each peak was assigned to the nearest TSS within 1 Mbp (Associating genomic regions with genes: ‘Single nearest gene’). The rank of GO terms was determined based on a hypergeometric test (‘Hyper rank’).

#### Comprehensive analysis of ChIP-seq and microarray data

Characterization of ChIP-seq signal profiles in non-overlapping 200-bp binned genomic regions was performed with chromHMM (numstates = 16) [64]. Briefly, this program separated the whole genomic region into 200-bp compartments. Next, the number of reads in each compartment is counted for 6 ChIP-seq data. The compartments which have similar data profile were combined and finally organized into 16 classes. To make the heatmap comprehensive, 2 classes which have almost no signals were combined into a class (class 15) since these classes were not used for further study. To evaluate the association between ChIP-seq and microarray data, first, a gene name was assigned to the compartments in each class if they were within 50 kbp around TSS (-25–+25 kbp) and make up a gene list for each class. Then, the genes in each microarray cluster were listed up. Finally, overlaps between these two gene lists were statistically evaluated by Fisher's exact test.

#### Correlation analysis of biological replicates for ChIP-seq data

The correlation of two biological replicates of ChIP-seq data was analyzed using DeepTools on a Galaxy platform [65]. Read counts of non-overlapping 10-kbp-binned genomic regions were calculated using multiBamSummary. Duplicated reads were ignored (‘ignoreDuplicates’).

### Luciferase reporter assay

Isolated human VWF promoter (-2182/+1475)-luc [66] was transiently co-transfected with pCI-Erg, pCI-Fli1, or both into Cos-7 cells. Two days later, luciferase activities were calculated using the Dual-Luciferase assay kit (Promega, Madison, WI, USA) as described previously [67].

### Co-immunoprecipitation

Cos-7 cells were transfected with Myc-tagged *Erg* (pEF6-*Erg*) and Flag-tagged *Fli1* (pFlag-CMV2-*Fli1*). After 24-hour incubation, cells were harvested and lysed with NP40 lysis buffer (0.5% NP-40, 50 mM Tris-HCl, 50 mM NaCl, 1 mM EDTA) containing complete protease inhibitor cocktail. After centrifuging at top speed for 10min, supernatant was precleared using ProteinG sepharose beads (71-7083-00 AI, GE healthcare), and then immunoprecipitation was performed with anti-Myc (5 μg; sc-40, Santa Cruz Biotechnology) or anti-Flag (5 μg; F1804, Sigma) overnight. After incubating with ProteinG beads for 2 hours, beads were washed with NP40 lysis buffer four times, boiled with Sample Buffer Solution with Reducing Reagent (09499–14, Nacalai), and then subjected to immunoblot analysis with anti-Myc-Tag (1:1000; 2278, Cell Signaling Technology) or anti-Flag (1:2000;14793, Cell Signaling Technology).

### Mice

C57BL6/N mice were purchased from Japan SLC (Shizuoka, Japan). All animals were housed under a 12-hour dark-light cycle at 22 ± 1°C with *ad libitum* food and water. The Animal Care and Use Committee of the University of Tokyo and The Animal Care and Use Committee of Kumamoto University School of Medicine approved the protocols for animal experiments. Male and female 5–8-week-old mice were used for the experiments.

### Tumor implantation

B16F10 cells (1 × 10^6^ cells) and 3LL-luc cells (1 × 10^6^ cells) were inoculated subcutaneously into the right flank of syngeneic C57BL/6N mice. E0771 cells (2 × 10^5^ cells) were inoculated into the mammary fat-pad of syngeneic female C57BL/6N mice. Seven days (B16F10) or 10 days (3LL-luc, E0771) after tumor inoculation, tumor tissues were used for immunofluorescent staining and collection of tumor tissue-conditioned media.

### Immunostaining

Mice were sacrificed with CO_2_ and perfused with 10 mL of 4% paraformaldehyde (09154–85, Nacalai). Tissues were harvested and fixed again with 4% paraformaldehyde for 2 hours at 4°C, followed by immersion in 30% sucrose overnight at 4°C. Tissues were embedded in OCT Compound (4583, Sakura Finetek Japan, Tokyo, Japan) and sectioned at a thickness of 10–20 μm with a Microm HM550 Cryostat (Thermo Fisher Scientific). The sections were blocked/permeabilized in 10% FBS/0.5% Triton X-100/PBS for 1 hour. The sections were incubated with anti-ERG (1:100; ab92513, Abcam), anti-FLI1 (1:100; ab15289, Abcam), and anti-CD31 (1:100; 550274, BD Biosciences) overnight at 4°C. The sections were then incubated with biotin-conjugated anti-rabbit IgG (1:1000; BA-1000, Vector Laboratories, Burlingame, CA, USA) overnight at 4°C. Finally, the sections were incubated with Alexa Fluor 488-conjugated streptavidin (S-32354, Thermo Fisher Scientific) and Alex Fluor 594-conjugated anti-rat IgG (A-21209, Thermo Fisher Scientific) overnight at 4°C. After the sections were incubated with TO-PRO-3 (1:500; T3650, Thermo Fisher Scientific) or DAPI (1:500; 342–07431, Dojindo, Kumamoto, Japan) for 20 minutes at room temperature, they were mounted with FluorSave Reagent (345789, Millipore). Samples were observed with a confocal laser microscope Fluoview FV-1000 (Olympus, Tokyo, Japan).

#### Tumor tissue-conditioned media

Tumor tissues were minced with surgical scissors and incubated for 40 minutes in HBSS containing 1 mg/mL collagenase type I (LS004196, Worthington, Lakewood, NJ, USA) and 100 μg/mL DNase I (11284932001, Roche) at 37°C. The digested tissues were further homogenized through wired mesh and then filtered through a Cell Strainer with a pore size of 40 μm (352340, Corning, Inc., Corning, NY, USA). Red blood cells were removed with ACK Lysing buffer (A1049201, Thermo Fisher scientific) for 1 minute at room temperature. Cells were incubated in 0.5% FBS/EBM-2 (CC-3156, Lonza) for 24 hours, and the media were used as tumor tissue-conditioned media. HUVECs were starved for 12–16 hours in 0.5% FBS/EBM-2, and subsequently treated with tumor tissue-conditioned medium for 4 or 24 hours. In control experiments, the cells were cultured in 0.5% FBS/EBM-2.

### Cell stimulation

#### Recombinant protein stimulation

HUVECs were treated with recombinant human FGF2 (100-18B, Peprotech, Rocky Hill, NJ, USA), IFNγ (093–05631, Wako), IL-1β (200-01B, Peprotech), TGFβ1 (100-21R, Peprotech), and TNFα (300-01A, Peprotech) at 10 ng/mL for 4 hours. In control experiments, a comparable amount of vehicle (PBS) was added.

#### CoCl_2_ stimulation

HUVECs were treated with 200 μg/mL cobalt(II) chloride hydrate (C8661, Sigma) for the indicated times. In control experiments, a comparable amount of vehicle (H_2_O) was added.

### Kaplan-Meier plot

Kaplan-Meier plots were obtained from the PrognoScan database [68]. Datasets listed in S13A Fig (Probe set ID: 213541_s_at) were used, and Kaplan-Meier plots were reconstituted based on the raw data table.

### Data accession

The accession number for the gene expression microarray and ChIP-seq reported in this paper is GEO: GSE109696. Other relevant data are within the paper and its Supporting Information files. Numerical data underlying all graphs in this manuscript is shown in S7 Table.

### Statistics

Data were analyzed by two-tailed unpaired Student’s *t*-test, non-parametric Mann-Whitney *U* test, or one-way ANOVA followed by Scheffe’s test. *P*-values < 0.05 were considered significant.

## Supporting information

S1 Fig Combined knockdown of *ERG* and *FLI1* induces EndMT, related to Fig 1. (A and B) Relative expression of *ERG*, *FLI1* (A), and endothelial/mesenchymal markers (B) quantified by qPCR in HUVECs treated with siERG, siFLI1, or both for 3 days. Another siRNA oligo set was used compared to the main figure. Data are represented as mean ± SEM (n = 3). *P < 0.05; **P < 0.01 by Student’s *t*-test. ND, not detected. (C) Morphology of HUVECs treated with siControl and siERG+siFLI1 at indicated time points after siRNA treatment. Scale bar, 150 μm. (D) Tube formation ability of HUVECs treated with siControl or siERG+siFLI1 for 7 days. Scale bar, 50 μm. (E) Immunoblot analysis of Caspase-3 in HUVECs treated with siERG, siFLI1, or both for 2 days. Arrowhead indicates cleaved Caspase-3. Signal intensity was quantified using the ImageJ software, and relative signal intensity (Cleaved Caspase-3/Non-cleaved Caspase-3) is shown. Data are represented as mean ± SEM (n = 3). **P < 0.01 by Student’s t-test.
(TIF)

S2 Fig Detailed analysis on gene expression microarray, related to Fig 1. (A) Z-score distribution is shown as violin plots in each cluster. Z-score was calculated using gene expression values in HUVECs treated with siERG, siFLI1, or both for 7 days. ^#^*P* < 0.01, ^##^*P* < 1×10^−5^, ^###^*P* < 1×10^−10^, ^####^*P* < 1×10^−15^; one-way ANOVA followed by Scheffe’s test. NS, not significant. (B) Heatmap of mRNA expression determined by microarray in HUVECs treated with siERG, siFLI1, or both for 3 days. EC-specific genes and mesenchymal-related genes are shown. (C) Gene ontology terms enriched in cluster 2 are listed.
(TIF)

S3 Fig Patterns of ups and downs in gene expression across 4 siRNA treatment groups, related to Fig 1. (A) Gene regulation patterns detected in day 3 datasets of Fig 1E. The ratio and number of genes coinciding with each pattern are shown as a pie chart and table. (B) Z-score distribution is shown as violin plots in each pattern. ^#^P < 0.01, ^##^P < 1×10^−5^, ^###^P < 1×10^−10^, ^####^P < 1×10^−15^; one-way ANOVA followed by Scheffe’s test.
(TIF)

S4 Fig ChIP-seq analysis of ERG and FLI1 in HUVECs, related to Fig 2. (A) Cross-reactivity of anti-ERG and anti-FLI1 were evaluated by immunoblot analysis. Cos-7 cells transfected with mouse *Erg* (Gene accession: NM_001302153.1) or *Fli1* (Gene accession: NM_008026) using pCI Mammalian Expression Vector (Promega, E1731) were used as samples. Anti-ERG [9FY] (ab136152) and Anti-FLI1 (ab15289) have specific binding activities against each target and thus were used for the ChIP assay, while immunohistochemistry analysis of ERG was performed with anti-ERG (ab92513) because ab136152 is not applicable to immunohistochemistry. (B) Reproducibility of ChIP-seq analysis assessed using two biological replicates. (C) Peak distributions of ERG and FLI1 around TSS.
(TIF)

S5 Fig ChIP-seq analysis of histone modifications in HUVECs, related to Fig 3. (A) Reproducibility of ChIP-seq analysis assessed using two biological replicates. (B) Heatmap showing H3K27me3 around ERG/FLI1-binding regions. (C) Peak distribution of H3K27me3 around ERG- and/or FLI1-binding regions. ChIP-seq data in (B) and (C) for H3K27me3 in HUVECs was obtained from ENCODE.
(TIF)

S6 Fig EndMT mediated by ERG/FLI1 loss is coupled to epigenetic changes, related to Fig 3. (A and B) ChIP-seq profiles of ERG, FLI1, and indicated histone modifications (siControl vs siERG+siFLI1) in HUVECs. Representative EC-specific gene loci (A) and mesenchymal- and inflammation-related gene loci (B) are shown.
(TIF)

S7 Fig Approach to search for a key EndMT regulator under the direct control of ERG and FLI1. (A and B) Flow chart shows our strategy to find a key EndMT regulator under the direct control of ERG and FLI1.
(TIF)

S8 Fig Possible role of TGFβ/SMAD signaling pathway. (A–B) ChIP-seq profiles of ERG, FLI1, and indicated histone modifications (siControl vs siERG+siFLI1) in HUVECs. *SMAD1* (A), *CNN1* and *TGFB2* (B) gene loci are shown. Relative expression of *SMAD1* (A), *CNN1* and *TGFB2* (B) quantified by gene expression microarray is also shown.
(TIF)

S9 Fig ChIP-seq data around the known EMT/EndMT-related microRNAs loci. ChIP-seq profiles of ERG, FLI1, and indicated histone modifications (siControl vs siERG+siFLI1) in HUVECs. Known EMT/EndMT-related microRNA loci are shown.
(TIF)

S10 Fig Loss of miR-126 partially induces EndMT, related to Fig 4. Relative expression of endothelial/mesenchymal markers quantified by qPCR in HUVECs treated with miR-126 inhibitor or control miRNA inhibitor for 3 days. Data are represented as mean ± SEM (n = 3). **P < 0.01 by Student’s t-test.
(TIF)

S11 Fig ERG and FLI1 expression is downregulated within ECs in E0771 and 3LL tumors, related to Fig 5. (A and B) Representative immunofluorescent staining of ECs in E0771 (A) and 3LL (B) tumor tissue. Immunofluorescent staining was reproduced in at least 3 independent mice. Arrows indicate ERG- or FLI1-negative ECs. Scale bar, 250 μm.
(TIF)

S12 Fig Inflammatory cytokines downregulate *ERG* and *FLI1* expression, related to Fig 6. (A) Relative expression of *ERG* and *FLI1* in HUVECs treated with the indicated recombinant proteins (10 ng/mL) for 4 hours. Data are represented as mean ± SEM (n = 3). *P < 0.05; **P < 0.01 by Student’s *t* test. NS, not significant. (B) Relative expression of *ERG* and *FLI1* in HUVECs treated with 250 μM CoCl_2_, a chemical inducer of hypoxia, for the indicated times. Data are represented as mean ± SEM (n = 3). *P < 0.05; **P < 0.01 by Student’s *t* test.
(TIF)

S13 Fig Clinical association between ERG expression and prognosis in cancer patients, related to Fig 7. (A) PrognoScan-based Kaplan-Meier plots of the indicated cancer types and endpoints. Probe set 213541_s_at was used for analysis. (B) Description of datasets shown in S13A Fig.
(TIF)

S14 Fig Exploration of downstream EndMT effectors after ERG and FLI1 knockdown. (A) Relative expression of *PIK3R2* quantified by qPCR. HUVECs were treated with siControl, siERG+siFLI1, or siERG+siFLI1+siSNAI2 for 3 days. (B and C) Relative expression of endothelial/mesenchymal markers quantified by qPCR. (B) HUVECs were treated with siControl, siERG+siFLI1, or siERG+siFLI1+siSNAI2 (two oligo sets) for 3 days. (C) HUVECs were treated with siControl, siERG+siFLI1, or siERG+siFLI1+siTWIST2 (two oligo sets) for 3 days. Data are represented as mean ± SEM (n = 3). *P < 0.05; **P < 0.01 by Student’s *t*-test. NS, not significant. ND, not detected.
(TIF)

S1 Table List of genes in each regulation pattern in S3 Fig. (XLSX)

S2 Table List of candidate coding genes responsible for regulating EndMT. (XLSX)

S3 Table Result of mirDIP target prediction. (XLSX)

S4 Table List of siRNAs and miRNAs. siERG#1 and siFLI1#1 were used throughout the study, while siERG#2 and siFLI1#2 were used in S1A and S1B Fig as an alternative set of siRNA oligos.
(XLSX)

S5 Table List of primers for qPCR. (XLSX)

S6 Table Summary of ChIP assay conditions. (XLSX)

S7 Table Numerical data underlying graphs in the manuscript. (XLSX)
