# Supplementary figures and images for: Downregulation of ERG and FLI1 expression in endothelial cells triggers endothelial-to-mesenchymal transition
Source: PLoS Genet. 2018 Nov 30;14(11):e1007826. doi: 10.1371/journal.pgen.1007826 (PMC6291168; doi:10.1371/journal.pgen.1007826)

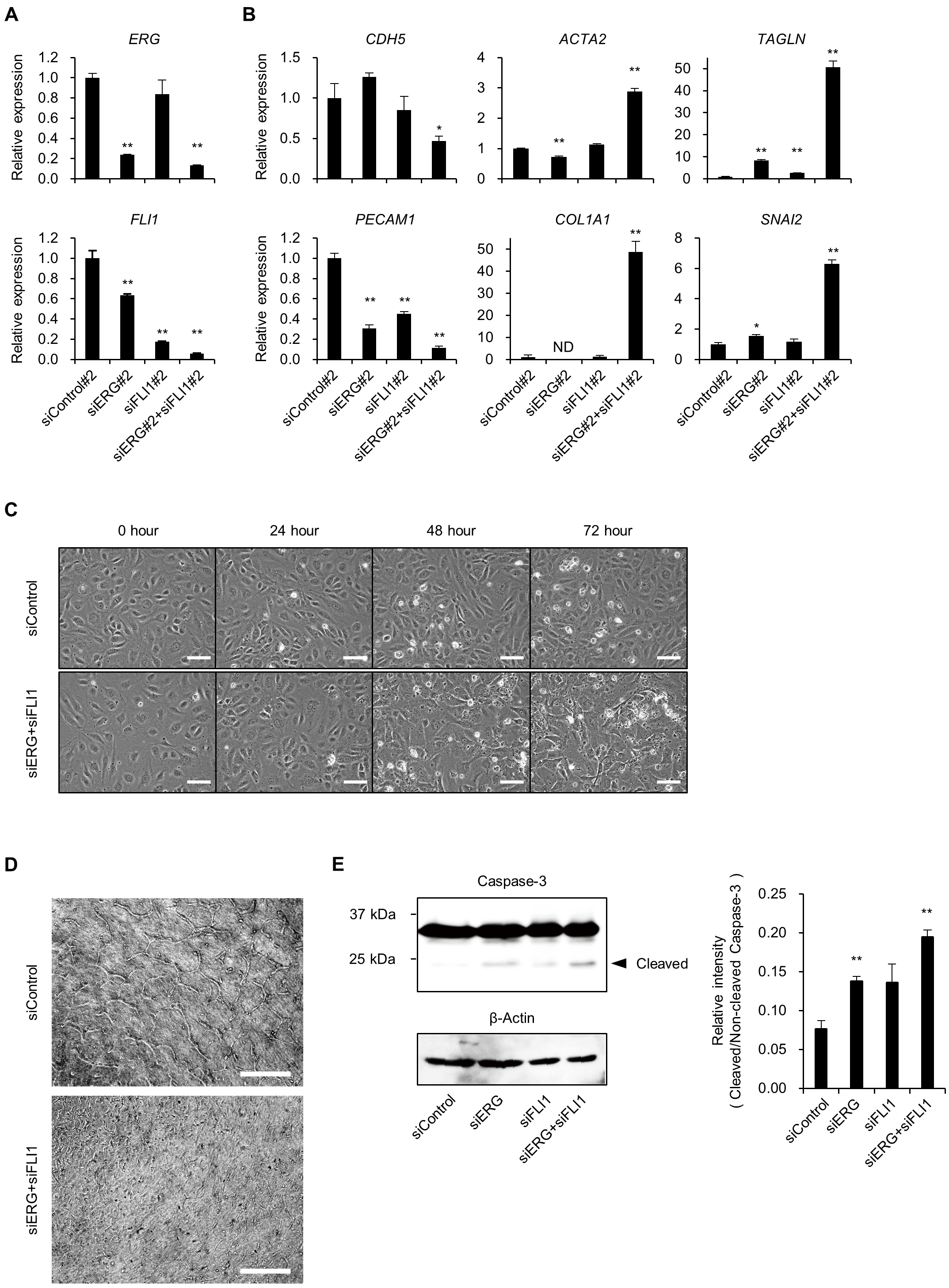

Supplement: S1 Fig — (A and B) Relative expression of ERG, FLI1 (A), and endothelial/mesenchymal markers (B) quantified by qPCR in HUVECs treated with siERG, siFLI1, or both for 3 days. Another siRNA oligo set was used compared to the main figure. Data are represented as mean ± SEM (n = 3). *P < 0.05; **P < 0.01 by Student’s t-test. ND, not detected. (C) Morphology of HUVECs treated with siControl and siERG+siFLI1 at indicated time points after siRNA treatment. Scale bar, 150 μm. (D) Tube formation ability of HUVECs treated with siControl or siERG+siFLI1 for 7 days. Scale bar, 50 μm. (E) Immunoblot analysis of Caspase-3 in HUVECs treated with siERG, siFLI1, or both for 2 days. Arrowhead indicates cleaved Caspase-3. Signal intensity was quantified using the ImageJ software, and relative signal intensity (Cleaved Caspase-3/Non-cleaved Caspase-3) is shown. Data are represented as mean ± SEM (n = 3). **P < 0.01 by Student’s t-test. (TIF) [file pgen.1007826.s001.tif]

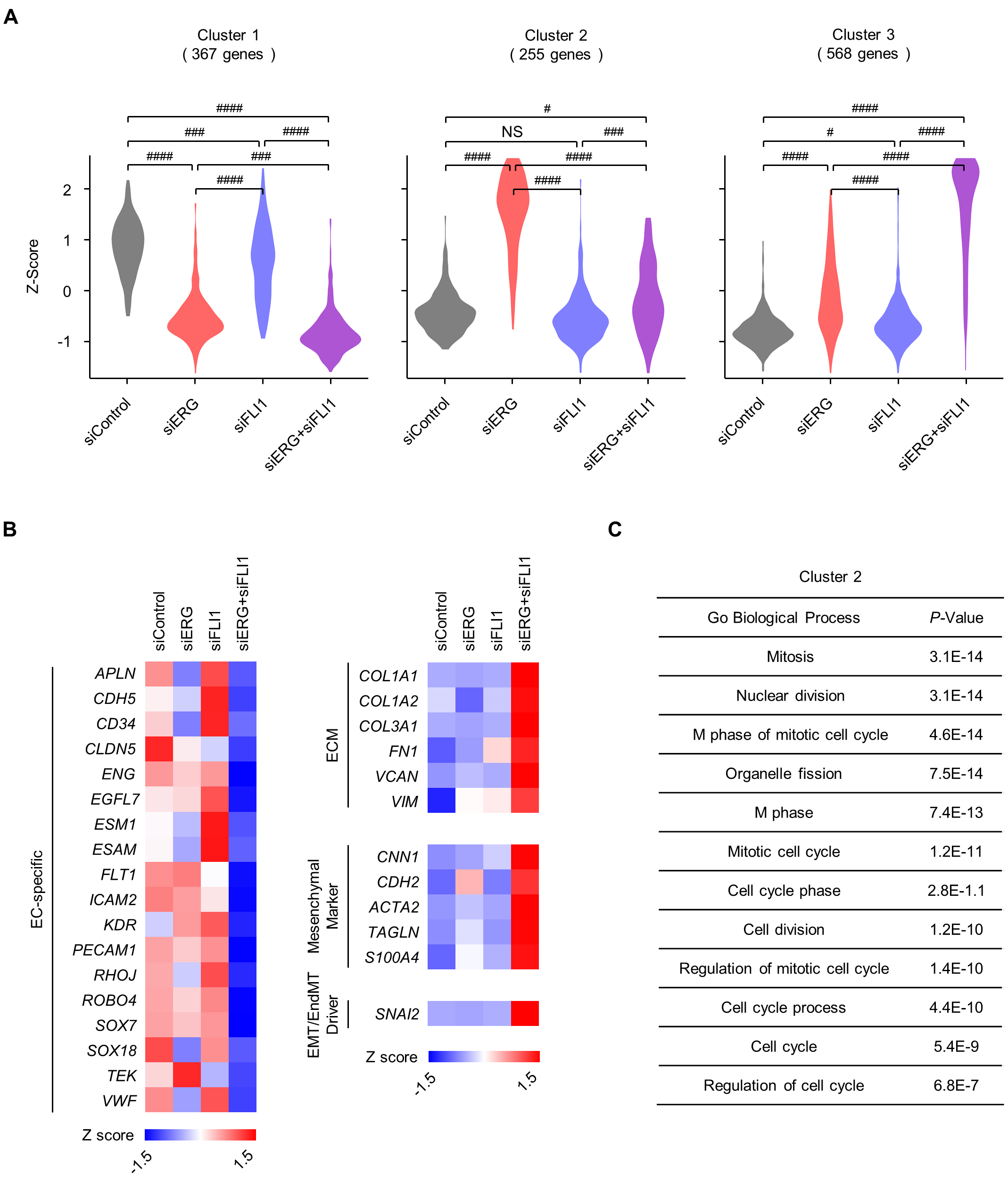

Supplement: S2 Fig — (A) Z-score distribution is shown as violin plots in each cluster. Z-score was calculated using gene expression values in HUVECs treated with siERG, siFLI1, or both for 7 days. #P < 0.01, ##P < 1×10^−5^, ###P < 1×10^−10^, ####P < 1×10^−15^; one-way ANOVA followed by Scheffe’s test. NS, not significant. (B) Heatmap of mRNA expression determined by microarray in HUVECs treated with siERG, siFLI1, or both for 3 days. EC-specific genes and mesenchymal-related genes are shown. (C) Gene ontology terms enriched in cluster 2 are listed. (TIF) [file pgen.1007826.s002.tif]

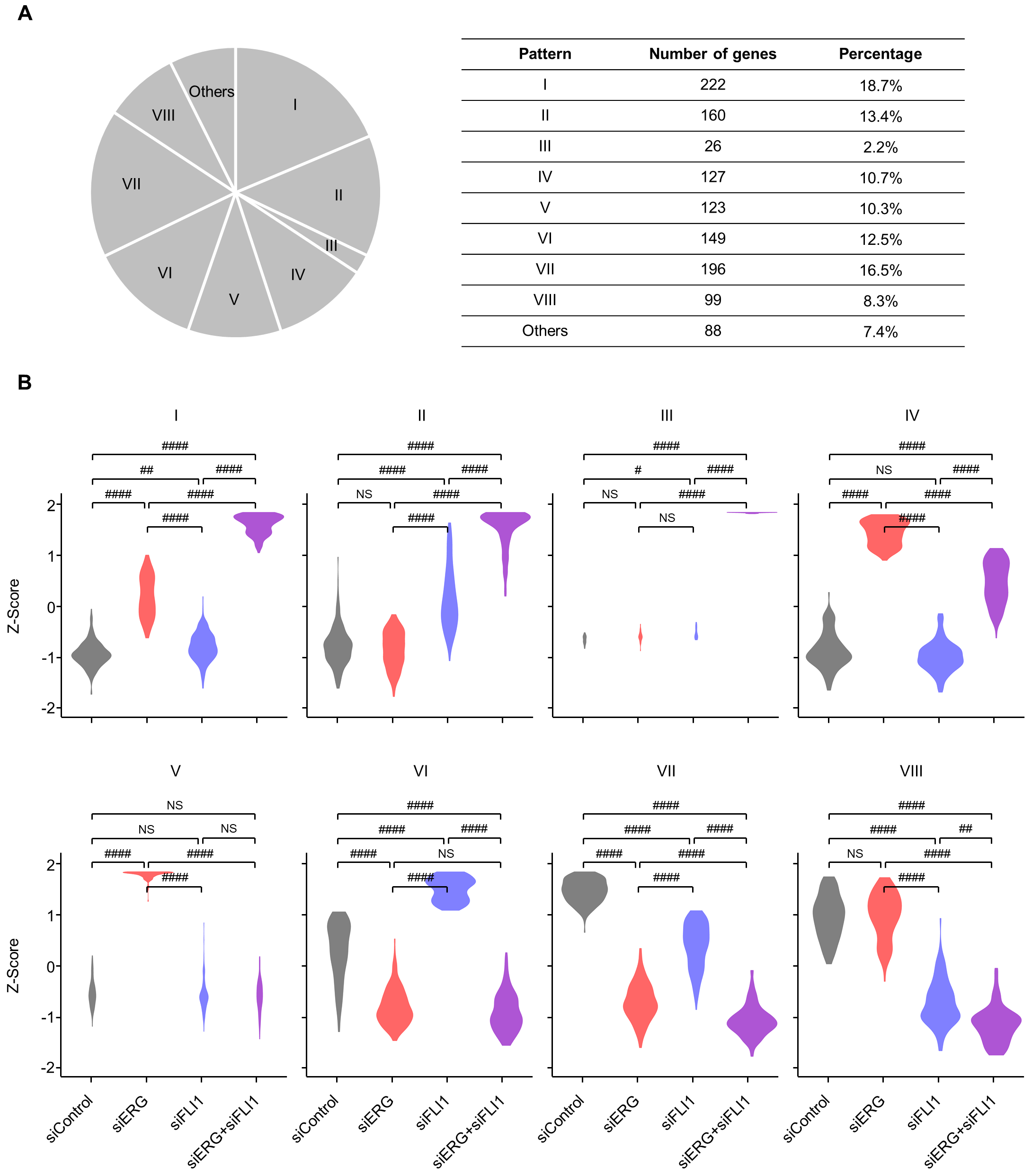

Supplement: S3 Fig — (A) Gene regulation patterns detected in day 3 datasets of Fig 1E. The ratio and number of genes coinciding with each pattern are shown as a pie chart and table. (B) Z-score distribution is shown as violin plots in each pattern. #P < 0.01, ##P < 1×10^−5^, ###P < 1×10^−10^, ####P < 1×10^−15^; one-way ANOVA followed by Scheffe’s test. (TIF) [file pgen.1007826.s003.tif]

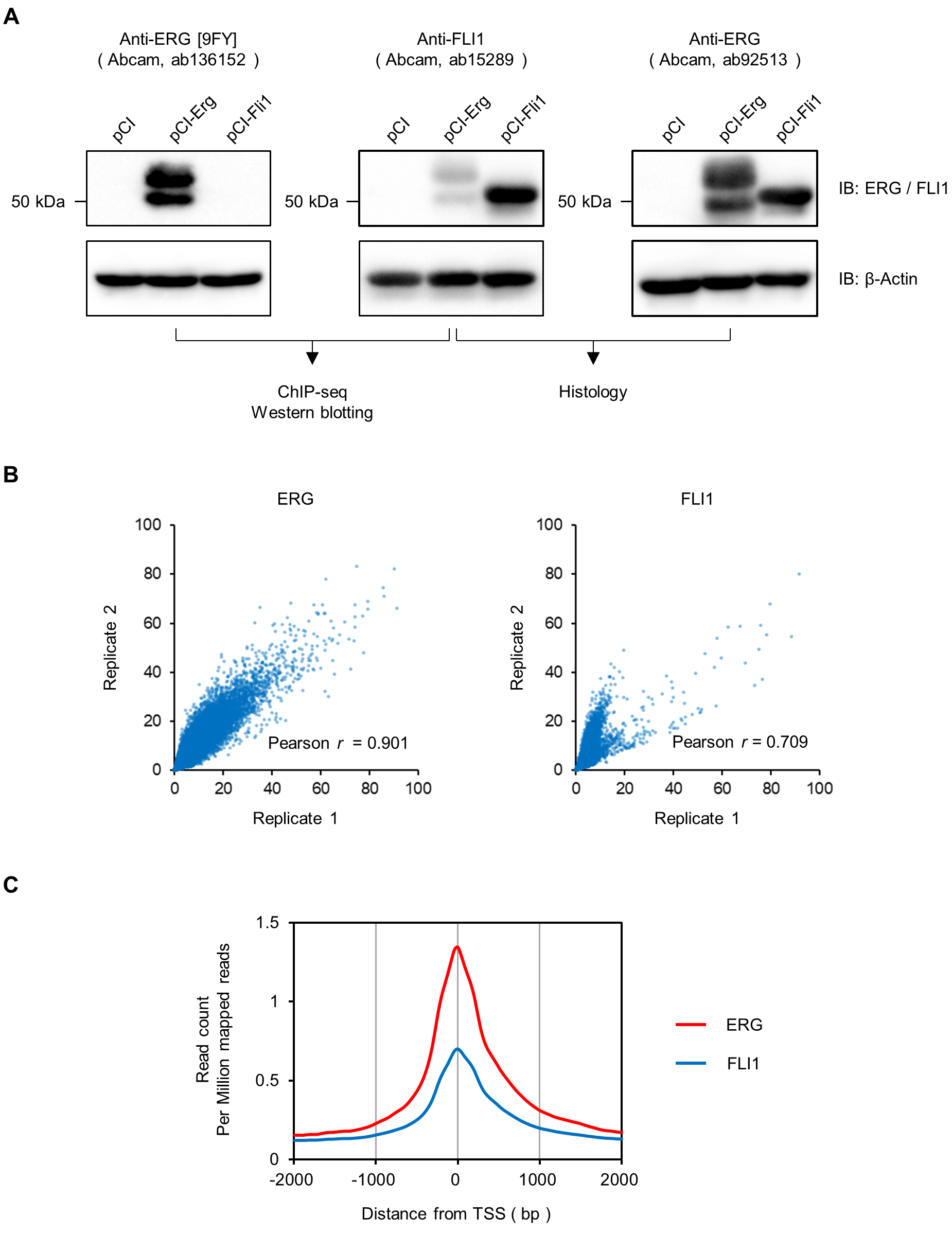

Supplement: S4 Fig — (A) Cross-reactivity of anti-ERG and anti-FLI1 were evaluated by immunoblot analysis. Cos-7 cells transfected with mouse Erg (Gene accession: NM_001302153.1) or Fli1 (Gene accession: NM_008026) using pCI Mammalian Expression Vector (Promega, E1731) were used as samples. Anti-ERG [9FY] (ab136152) and Anti-FLI1 (ab15289) have specific binding activities against each target and thus were used for the ChIP assay, while immunohistochemistry analysis of ERG was performed with anti-ERG (ab92513) because ab136152 is not applicable to immunohistochemistry. (B) Reproducibility of ChIP-seq analysis assessed using two biological replicates. (C) Peak distributions of ERG and FLI1 around TSS. (TIF) [file pgen.1007826.s004.tif]

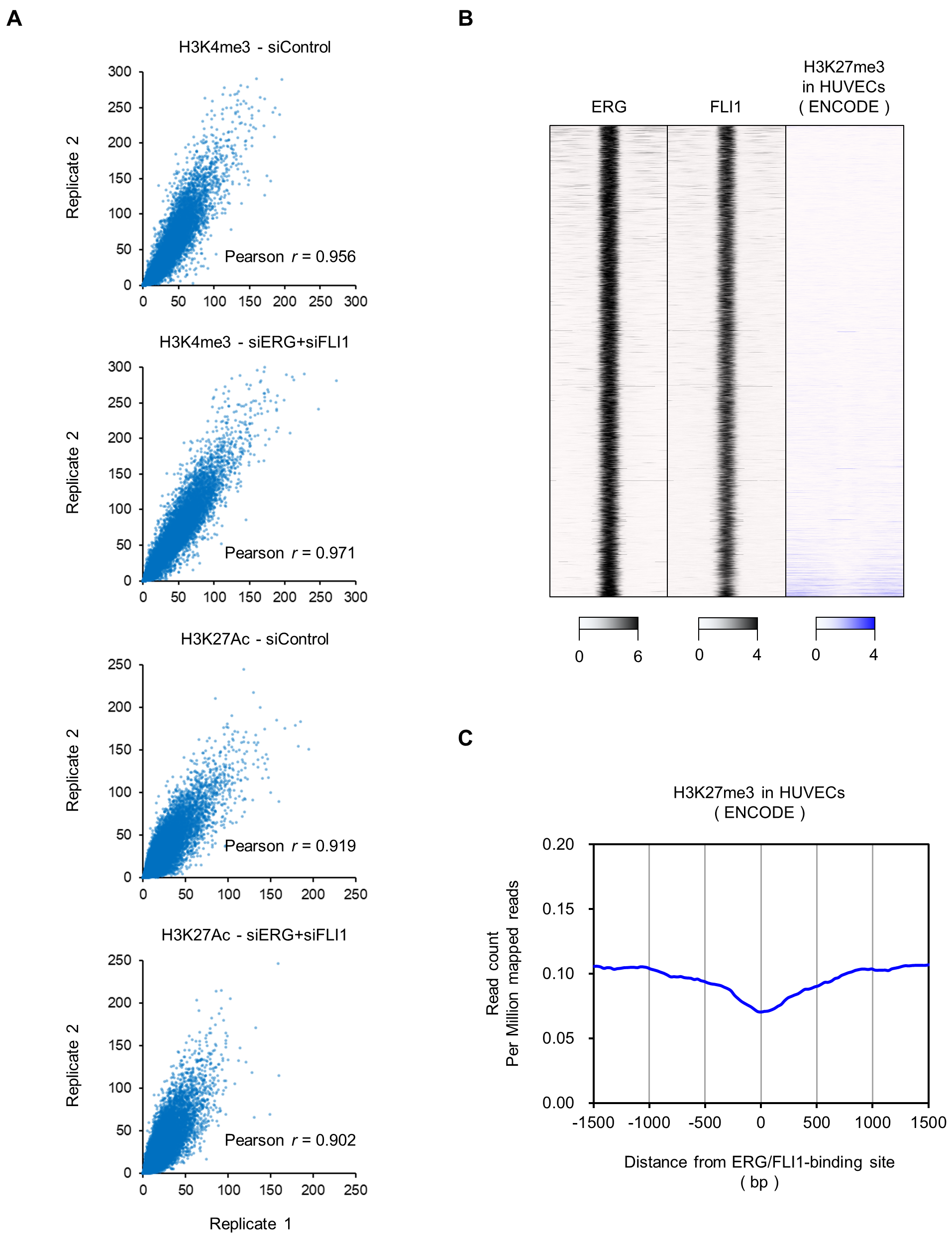

Supplement: S5 Fig — (A) Reproducibility of ChIP-seq analysis assessed using two biological replicates. (B) Heatmap showing H3K27me3 around ERG/FLI1-binding regions. (C) Peak distribution of H3K27me3 around ERG- and/or FLI1-binding regions. ChIP-seq data in (B) and (C) for H3K27me3 in HUVECs was obtained from ENCODE. (TIF) [file pgen.1007826.s005.tif]

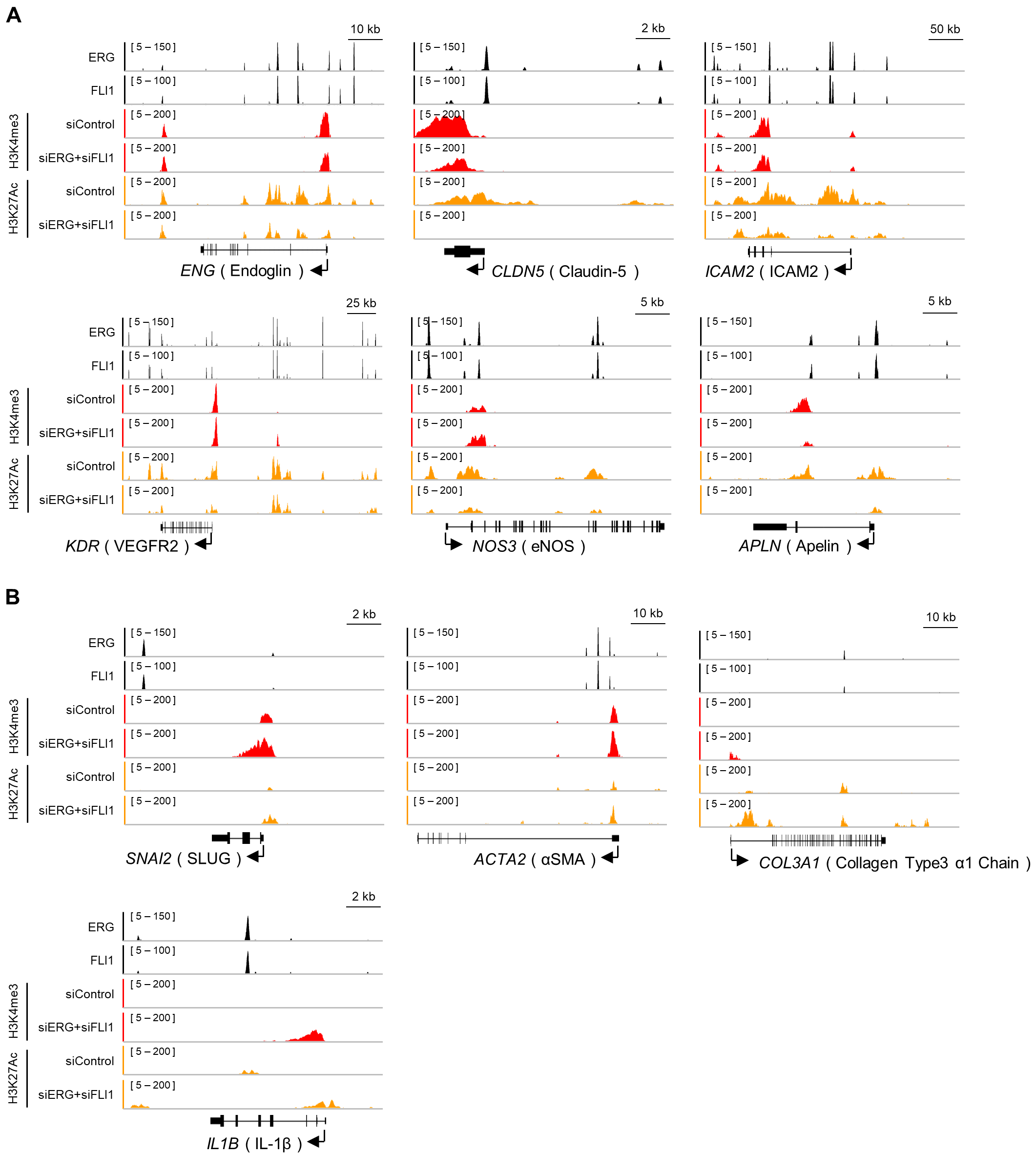

Supplement: S6 Fig — (A and B) ChIP-seq profiles of ERG, FLI1, and indicated histone modifications (siControl vs siERG+siFLI1) in HUVECs. Representative EC-specific gene loci (A) and mesenchymal- and inflammation-related gene loci (B) are shown. (TIF) [file pgen.1007826.s006.tif]

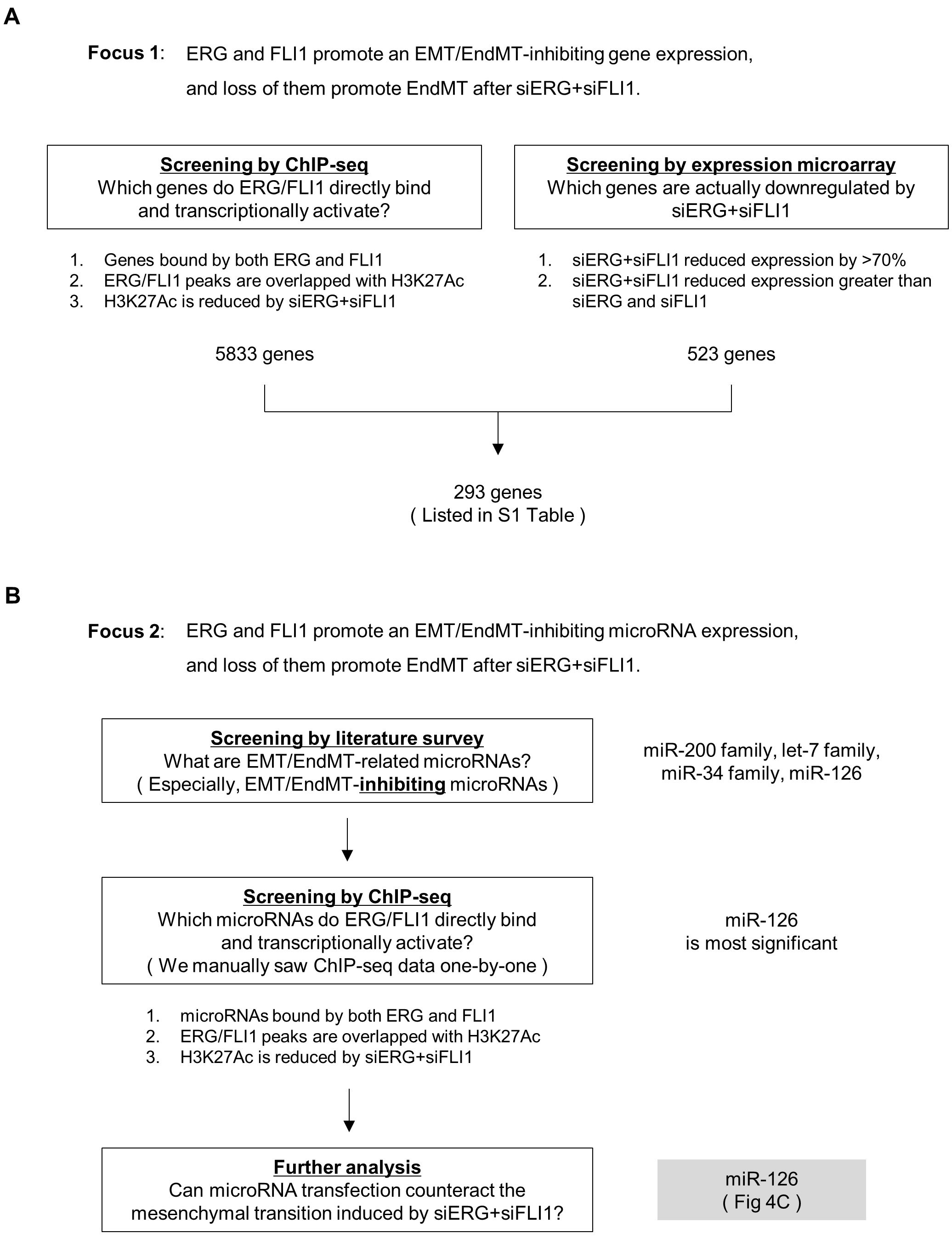

Supplement: S7 Fig — (A and B) Flow chart shows our strategy to find a key EndMT regulator under the direct control of ERG and FLI1. (TIF) [file pgen.1007826.s007.tif]

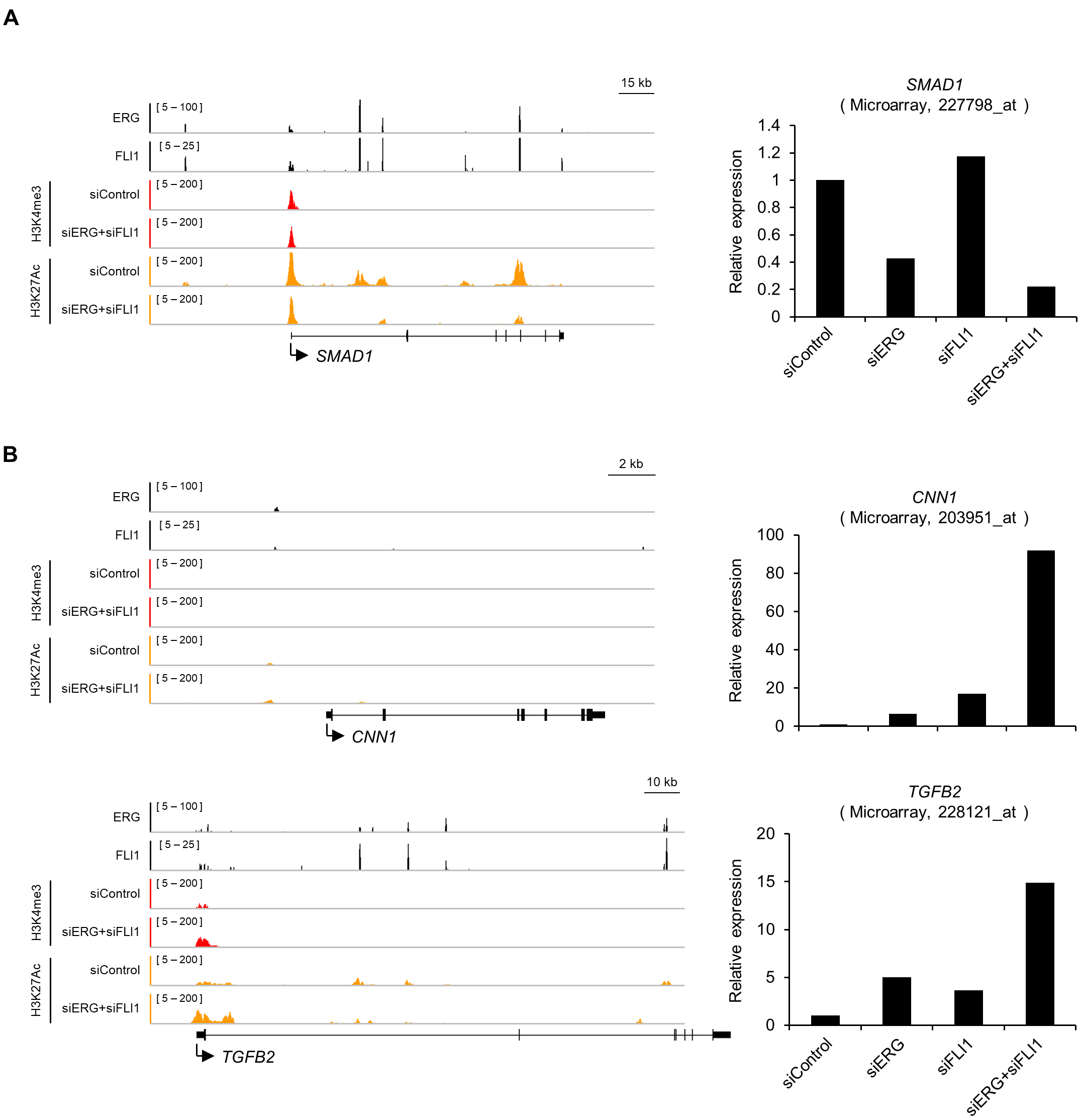

Supplement: S8 Fig — (A–B) ChIP-seq profiles of ERG, FLI1, and indicated histone modifications (siControl vs siERG+siFLI1) in HUVECs. SMAD1 (A), CNN1 and TGFB2 (B) gene loci are shown. Relative expression of SMAD1 (A), CNN1 and TGFB2 (B) quantified by gene expression microarray is also shown. (TIF) [file pgen.1007826.s008.tif]

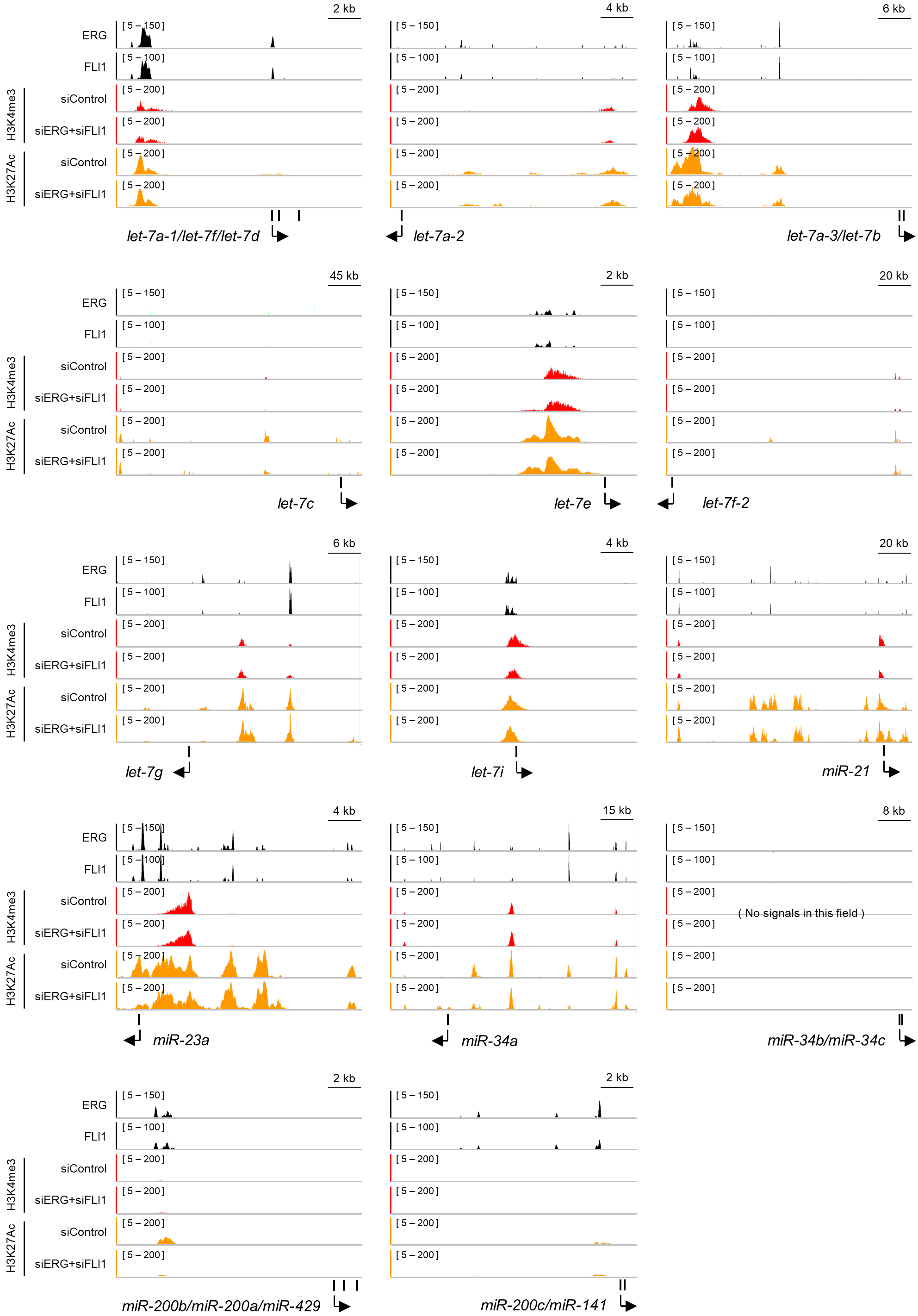

Supplement: S9 Fig — ChIP-seq profiles of ERG, FLI1, and indicated histone modifications (siControl vs siERG+siFLI1) in HUVECs. Known EMT/EndMT-related microRNA loci are shown. (TIF) [file pgen.1007826.s009.tif]

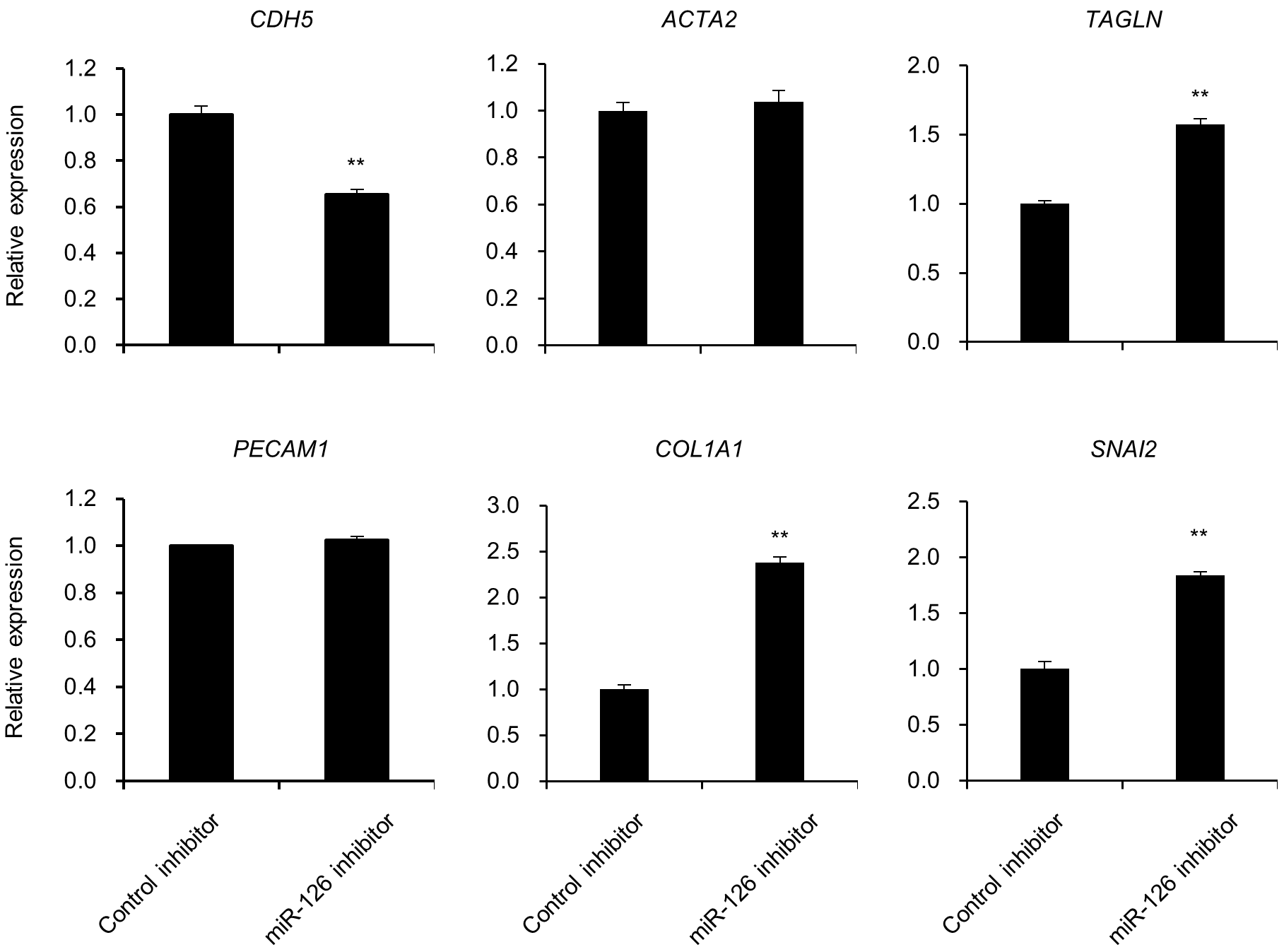

Supplement: S10 Fig — Relative expression of endothelial/mesenchymal markers quantified by qPCR in HUVECs treated with miR-126 inhibitor or control miRNA inhibitor for 3 days. Data are represented as mean ± SEM (n = 3). **P < 0.01 by Student’s t-test. (TIF) [file pgen.1007826.s010.tif]

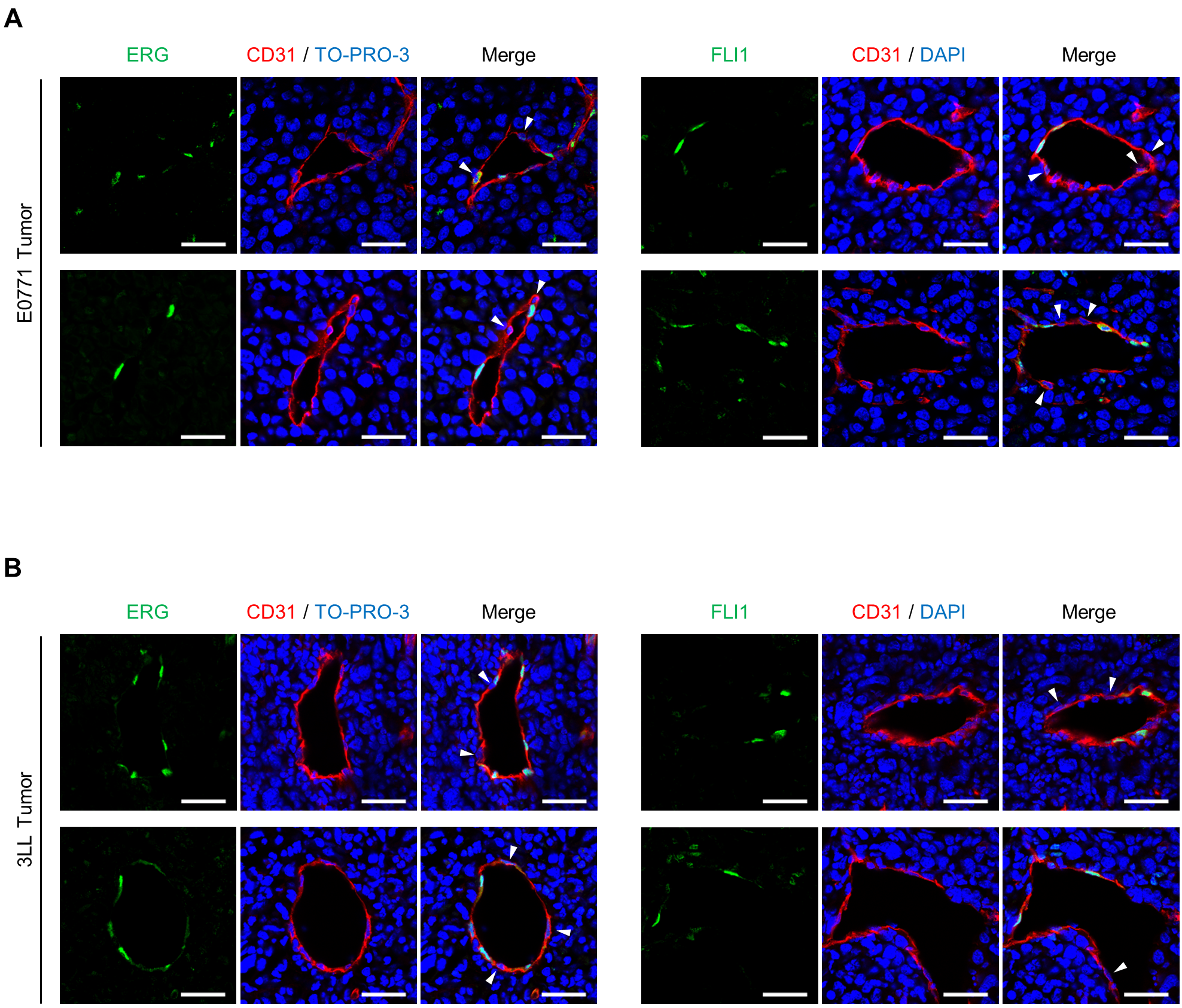

Supplement: S11 Fig — (A and B) Representative immunofluorescent staining of ECs in E0771 (A) and 3LL (B) tumor tissue. Immunofluorescent staining was reproduced in at least 3 independent mice. Arrows indicate ERG- or FLI1-negative ECs. Scale bar, 250 μm. (TIF) [file pgen.1007826.s011.tif]

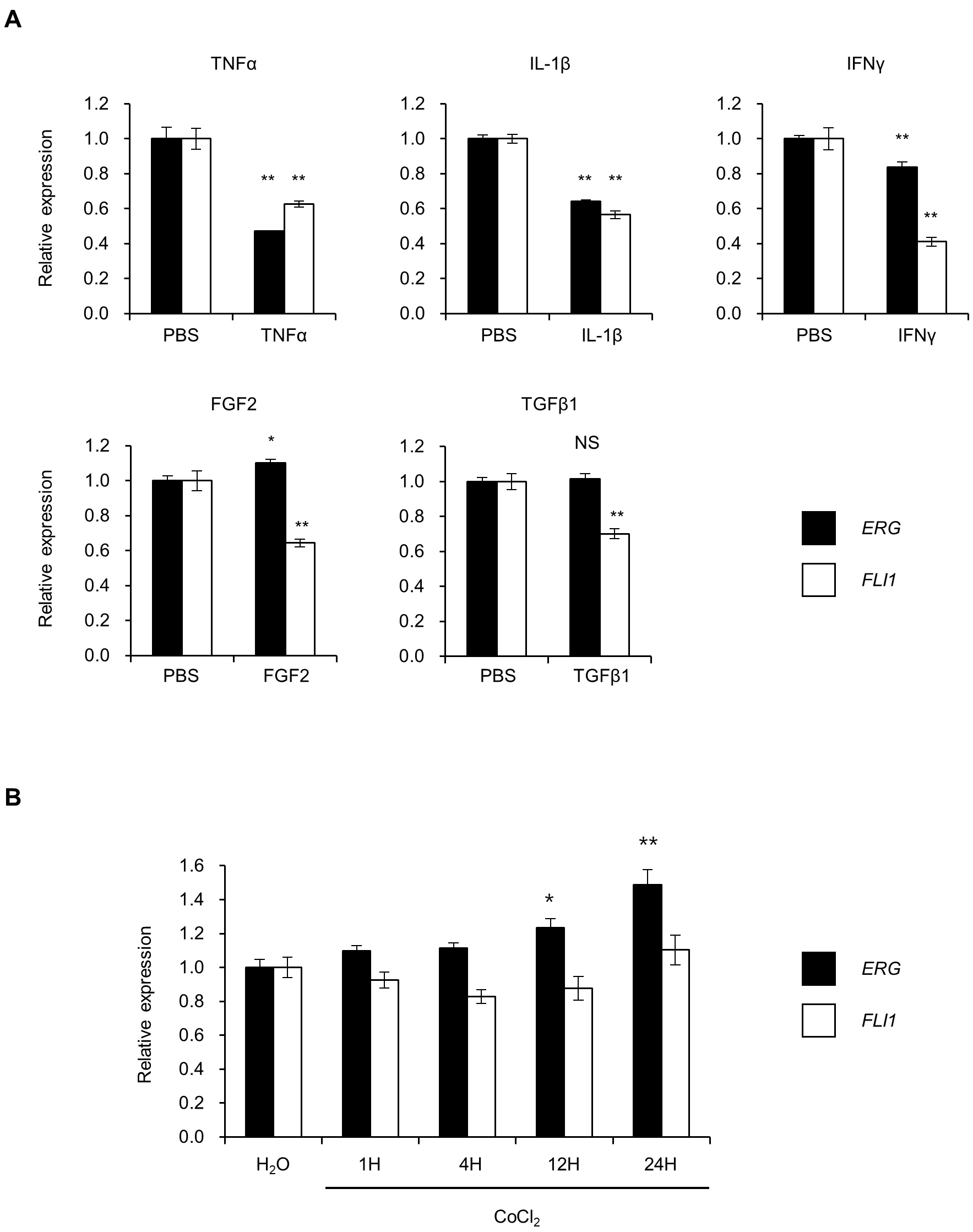

Supplement: S12 Fig — (A) Relative expression of ERG and FLI1 in HUVECs treated with the indicated recombinant proteins (10 ng/mL) for 4 hours. Data are represented as mean ± SEM (n = 3). *P < 0.05; **P < 0.01 by Student’s t test. NS, not significant. (B) Relative expression of ERG and FLI1 in HUVECs treated with 250 μM CoCl2, a chemical inducer of hypoxia, for the indicated times. Data are represented as mean ± SEM (n = 3). *P < 0.05; **P < 0.01 by Student’s t test. (TIF) [file pgen.1007826.s012.tif]

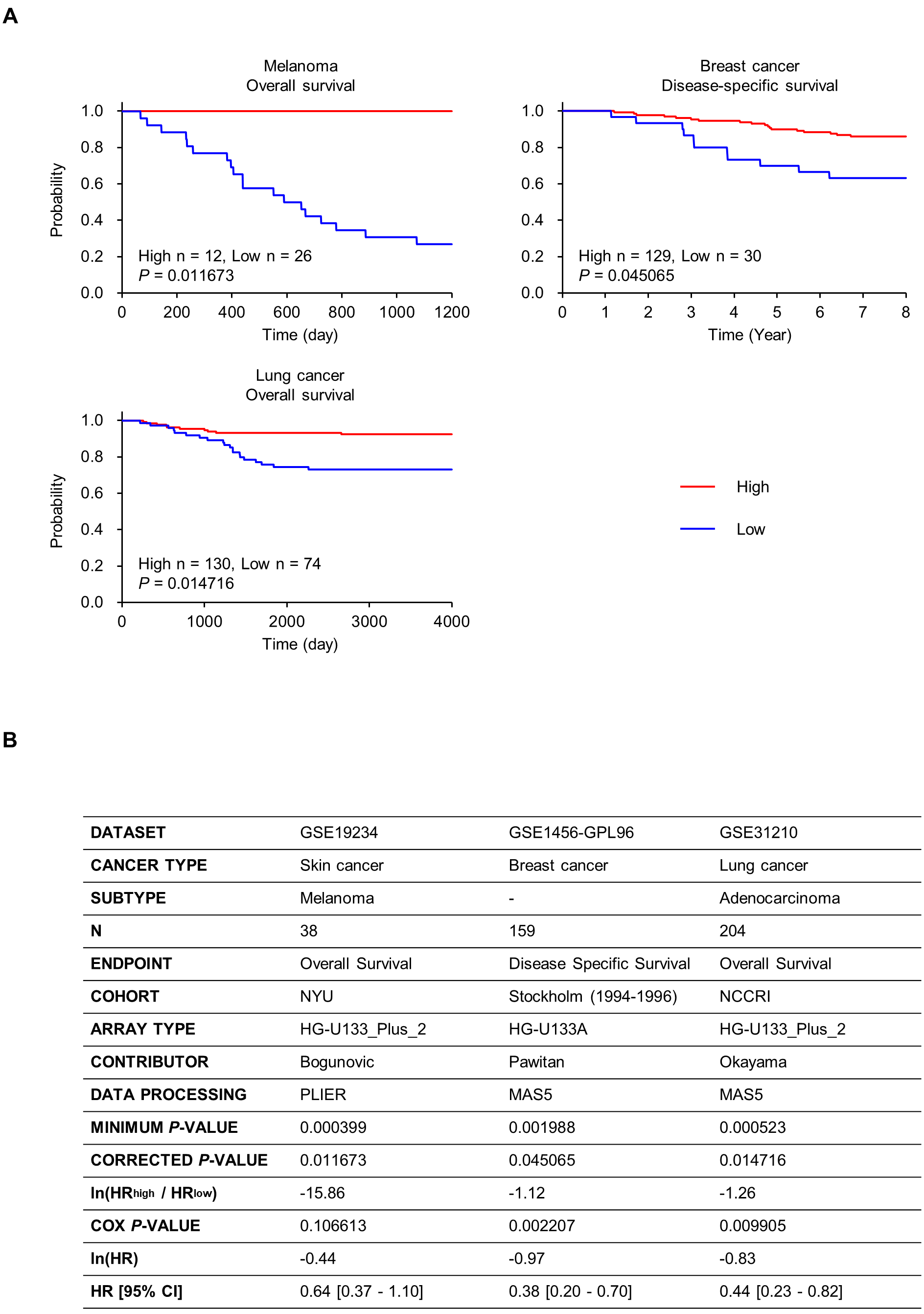

Supplement: S13 Fig — (A) PrognoScan-based Kaplan-Meier plots of the indicated cancer types and endpoints. Probe set 213541_s_at was used for analysis. (B) Description of datasets shown in S13A Fig. (TIF) [file pgen.1007826.s013.tif]

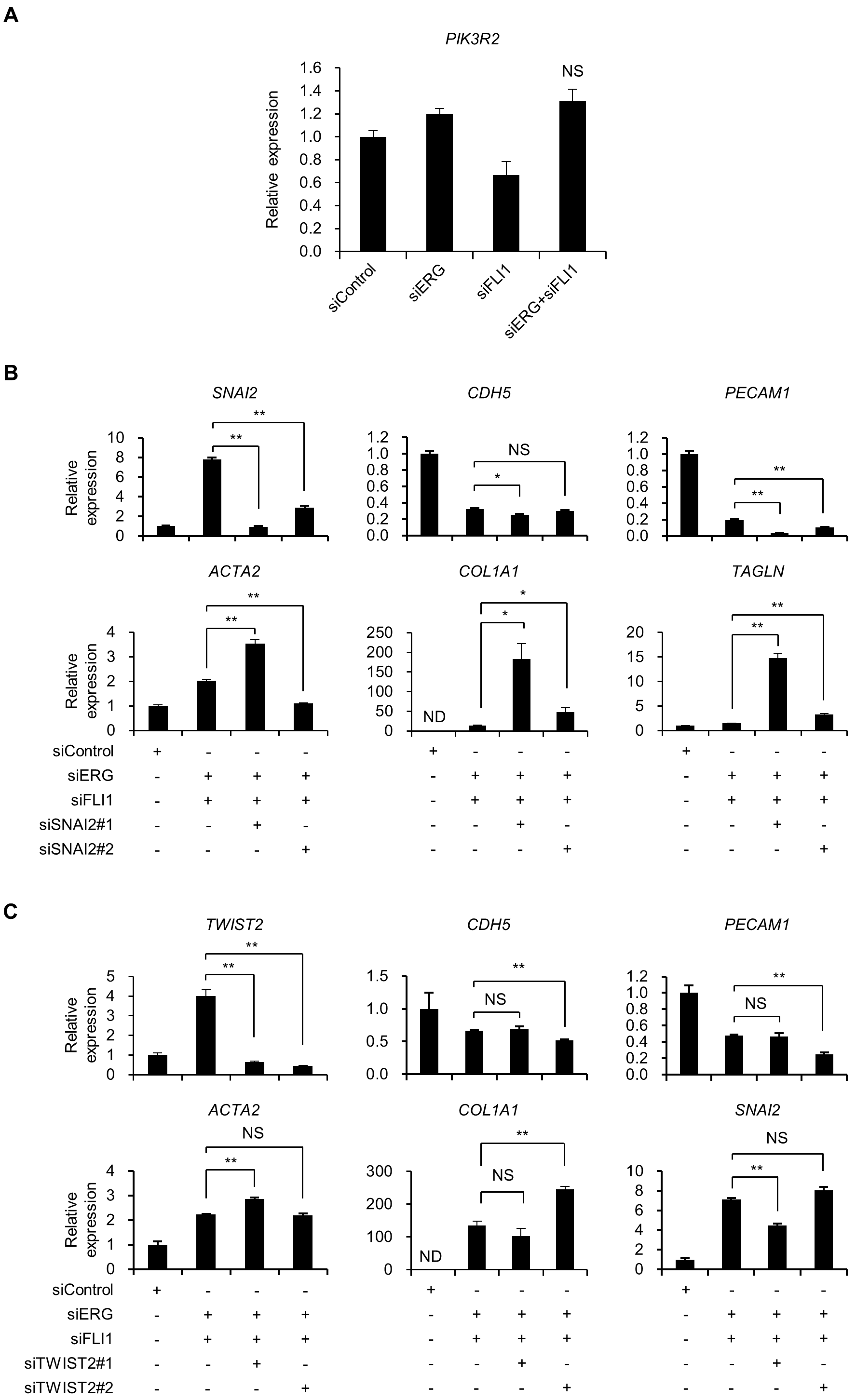

Supplement: S14 Fig — (A) Relative expression of PIK3R2 quantified by qPCR. HUVECs were treated with siControl, siERG+siFLI1, or siERG+siFLI1+siSNAI2 for 3 days. (B and C) Relative expression of endothelial/mesenchymal markers quantified by qPCR. (B) HUVECs were treated with siControl, siERG+siFLI1, or siERG+siFLI1+siSNAI2 (two oligo sets) for 3 days. (C) HUVECs were treated with siControl, siERG+siFLI1, or siERG+siFLI1+siTWIST2 (two oligo sets) for 3 days. Data are represented as mean ± SEM (n = 3). *P < 0.05; **P < 0.01 by Student’s t-test. NS, not significant. ND, not detected. (TIF) [file pgen.1007826.s014.tif]
